# Molecular design, synthesis and biological evaluation of novel 1,2,5-trisubstituted benzimidazole derivatives as cytotoxic agents endowed with ABCB1 inhibitory action to overcome multidrug resistance in cancer cells

**DOI:** 10.1080/14756366.2022.2127700

**Published:** 2022-09-27

**Authors:** Abeer H. A. Abdelhafiz, Rabah A. T. Serya, Deena S. Lasheen, Nessa Wang, Mansour Sobeh, Michael Wink, Khaled A. M. Abouzid

**Affiliations:** a Pharmaceutical Chemistry Department, Faculty of Pharmacy, Ain Shams University, Abbassia, Cairo, Egypt; b Institute of Pharmacy and Molecular Biotechnology, Heidelberg University, Heidelberg, Germany; c AgroBioSciences Research, Mohammed VI Polytechnic University, Ben-Guerir, Morocco

**Keywords:** Multidrug resistnce (MDR), ABCB1 transporter, benzimidazole, ligand based pharmacophore, molecular modelling

## Abstract

Multidrug resistance (MDR) is a leading cause for treatment failure in cancer patients. One of the reasons of MDR is drug efflux by ATP-binding cassette (ABC) transporters in eukaryotic cells especially ABCB1 (P-glycoprotein). In this study, certain novel 1,2,5-trisubstituted benzimidazole derivatives were designed utilising ligand based pharmacophore approach. The designed benzimidazoles were synthesised and evaluated for their cytotoxic activity towards doxorubicin-sensitive cell lines (CCRF/CEM and MCF7), as well as against doxorubicin-resistant cancer cells (CEM/ADR 5000 and Caco-2). In particular, compound **VIII** showed a substantial cytotoxic effect in all previously mentioned cell lines especially in doxorubicin-resistant CEM/ADR5000 cells (IC_50_ = 8.13 µM). Furthermore, the most promising derivatives **VII**, **VIII** and **XI** were tested for their ABCB1 inhibitory action in the doxorubicin-resistant CEM/ADR 5000 subline which is known for overexpression of ABCB1 transporters. The results showed that compound **VII** exhibited the best ABCB1 inhibitory activity at three tested concentrations (22.02 µM (IC_50_), 50 µM and 100 µM) in comparison to verapamil as a reference ABCB1 inhibitor. Such inhibition resulted in a synergistic effect and a massive decrease in the IC_50_ of doxorubicin (34.5 µM) when compound **VII** was used in a non-toxic dose in combination with doxorubicin in doxorubicin-resistant cells CEM/ADR 5000 (IC_50(Dox+VII)_ = 3.81 µM). Molecular modelling studies were also carried out to explain the key interactions of the target benzimidazoles at the ABCB1 binding site. Overall the obtained results from this study suggest that 1,2,5-trisubstituted benzimidazoles possibly are promising candidates for further optimisation and development of potential anticancer agents with ABCB1 inhibitory activity and therefore overcome MDR in cancer cells.

## Graphical Abstract

**Figure:**
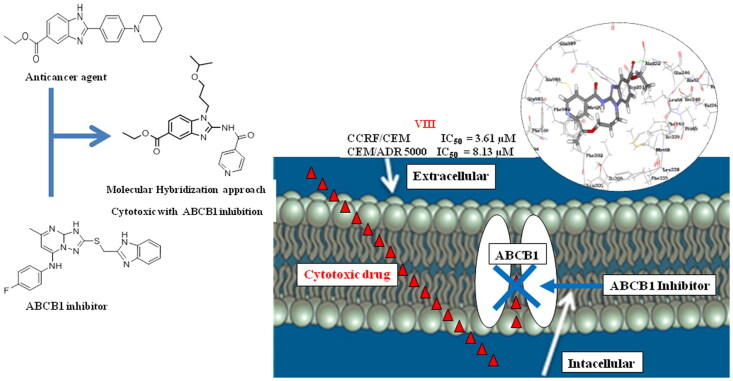

## Introduction

MDR may develop in cancer cells during treatment for different cytotoxic drugs that are structurally or functionally unrelated^1^^,^^2^ leading to drug failure of several anticancer agents^3^. Three possible mechanisms are involved in MDR including decreased cellular uptake of hydrophilic drug, decreased apoptotic activity which enhances the cell survival and increased drug efflux by ATP binding cassette (ABC) transporters^4^. Such transporters are responsible for the drug efflux thus decreasing the intracellular concentration of the cytotoxic drug^5^ and hence tumour cells are protected against the toxic effects of the anticancer agent^6^.

**Figure 1:**
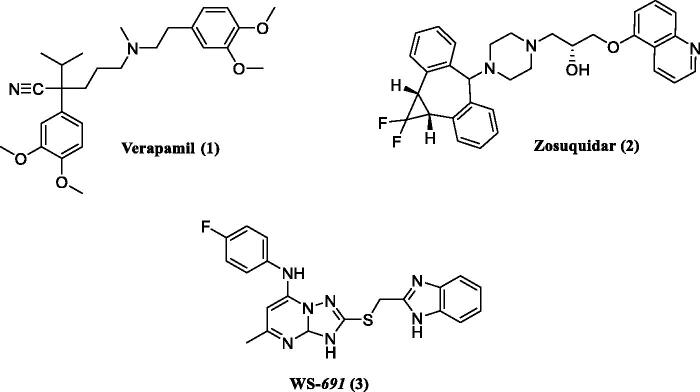
Chemical structures of ABCB1 inhibitors.

To our knowledge, there are 50 human ABC transporters genes encoding the formation of membrane-bound pumps which bind and transport a diverse set of substrates^7^. Among the various MDR-related ABC transporters, the most prominent members are ABCB1^4^, ABCC1 and ABCG2^8^^,^^9^. ABCB1 drug transporter (also known as P-glycoprotein or MDR1) is a well-studied and characterised member of the ABCB superfamily, which is responsible for the efflux of cytotoxic drugs thus decreasing their intracellular concentration^10^. The first generation ABCB1 inhibitor, verapamil (**1**) (Figure 1), was reported to overcome P-glycoprotein mediated drug efflux of Vinca alkaloids. However, it was never used clinically to overcome MDR due to its devastating side effects as nausea, vomiting and abdominal pain^11^. More recently developed ABCB1 inhibitors as zosuquidar (**2**) showed less adverse effects, but no improvement in the treatment was recorded during clinical trials^12^. So far, various possible reasons contribute to the failure of these tested ABCB1 inhibitors. Lack of specificity and severe adverse effects are the suggested main contributors to the failure of such inhibitors^13^. Interestingly, a recent study reported the discovery of a new ABCB1 inhibitor WS-*691* (**3**) which was used in combination with PTX (paclitaxel) in PTX-resistant cells where ABCB1 are overexpressed. The use of a non-toxic dose of WS-*691* (20 µM) successfully decreased the IC_50_ of PTX from 4.23 to 0.022 µM without evidence to exhibit cytotoxic activity when used alone^10^. Remarkably, ABCB1 transporters are ubiquitously overexpressed in many MDR cancer cells^14–16^. Consequently, ABCB1 inhibition is a rewarding target to overcome failure of cytotoxic agents due to MDR.

**Figure 2.**
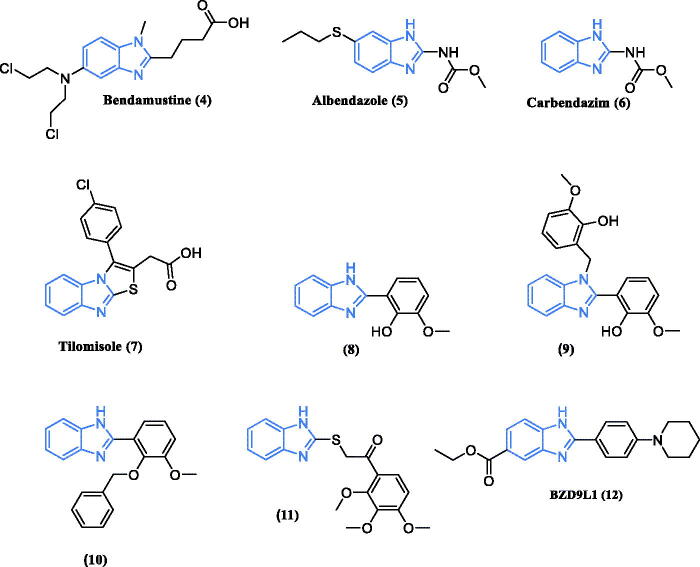
Chemical structures of benzimidazole based compounds with anticancer activity.

Benzimidazole is a privileged scaffold which is incorporated in various bioactive compounds that reveal a wide range of biological activities and commonly used as anticancer, antihypertensive, antiviral, antifungal and anti-HIVs agents^17^. The benzimidazole scaffold is frequently used in many anticancer agents^18^^,^^19^ (Figure 2) such as bendamustine (**4**)^20^. Also, several benzmidazole based compounds exhibited an anticancer activity such as albendazole (**5**)^21^, carbendazim (**6**)^22^, tilomisole (Wy-18,251) (**7**)^23^. Moreover, some 2-substituted benzimidazole derivatives such as (**8–10**)^24^, (**11**)^25^ and (**12**)^26^ displayed a well tolerated anticancer activity in the cytotoxicity assays. Remarkably, the potent antiproliferative activity of the 2-(benzimidazol-2-yl)thio derivative (**11**) was apparent against colon cancer cells^25^. Interestingly, the 2,5-disubstituted benzimidazole derivative BD9L1 (**12**) displayed a substantial antiproliferative effects across a panel of cancer cell lines mainly CCRF/CEM leukaemia cells^26^.

## Rationale and design

**Figure 3.**
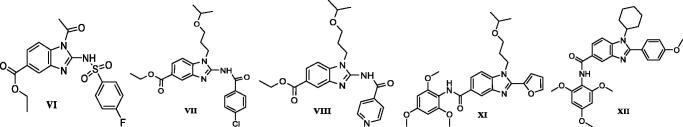
Chemical structures of proposed benzimidazole compounds (**VI-VIII, XI and XII**).

**Figure 4.**
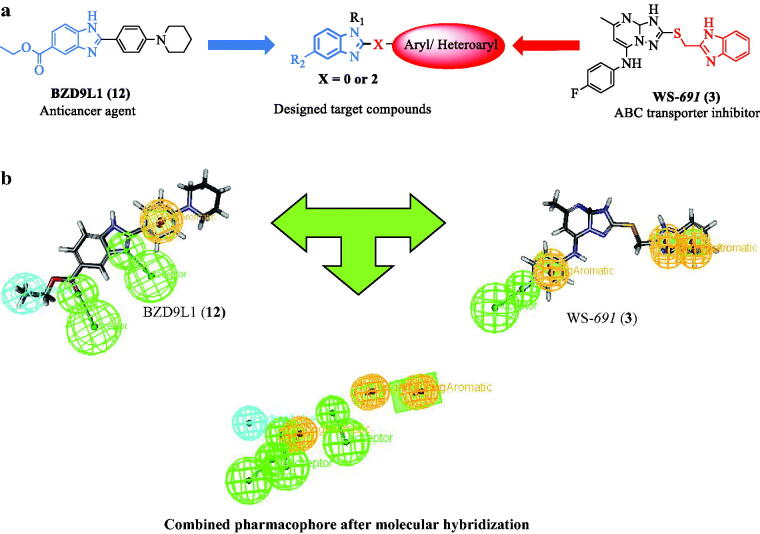
**(a)** Schematic illustration of the proposed target compounds scaffolds based on the chemical structures of the benzimidazole based inhibitor BZD9L1 (**12**) as a potent anticancer agent and WS-*691* (**3**) as ABCB1 inhibitor. **(b)** Molecular hybridisation and ligand based pharmacophore generation based on the essential features in anticancer BZD9L1 (**12**) and ABCB1 inhibitor WS-*691* (**3**) as reported. The green colour indicated H-**b**ond acceptor feature, Cyan colour indicates hydrophobic feature and orange colour indicates ring aromatic feature.

This work aims to design and synthesise novel 1,2,5-trisubstituted benzimidazole derivatives (**VI-VIII, XI** and **XII**) for both evaluation of their anticancer and ABCB1 inhibitory activities (Figure 3). The design of the target compounds was achieved by molecular hybridisation of the essential pharmacophoric features of 2,5-disubstituted benzimidazole anticancer compound BZD9L1 (**12**) and the orally active ABCB1 inhibitor WS-*691* (**3**) (Figure 4(a,b)). Our design of the target compounds comprised the substitution of all derivatives at the 2-position of the benzimidazole core mimicking the substitution at 2-position of triazolopyrimidine scaffold in WS-*691* (**3**) since it is essential for ABCB1 inhibition as reported^10^. The substitution at the 2-position was either *via* 2 atom spacer (**VI-VIII**) or directly attached to the benzimidazole scaffold (**XI** and **XII**) in order to evaluate the effects of such alteration in the structure on the ABCB1 inhibition. The design also involved the replacement of the 5-carboxylate moiety in BZD9L1 (**12**) with an amide group as a bioisostere in the proposed compounds (**XI** and **XII**) to evaluate the effect of this replacement on the anticancer and the ABCB1 inhibitory activities of our target compounds as well. Finally, aliphatic acyclic or cyclic chain (**VI-VIII, XI and XII**) was introduced at position 1 of the benzimidazole ring to further study the effect of this modification on the biological activity. Moreover, the suggested derivatives were subjected to ligand pharmacophore mapping over the combined pharmacophore built up from the molecular hybridisation of both reference compounds WS-*691* (**3**) and BZD9L1 (**12**). This was performed using ligand pharmacophore mapping protocol, Accelrys Discovery Studio® 2.5.5 software. Interestingly, the results showed a well-fitting of our proposed compounds. These promising results supported our design strategy of the proposed benzimidazole derivatives for their synthesis and subsequent evaluation of their anticancer activity as well as their ability to modulate ABCB1 transporters thus overcome multidrug resistance in caner cells.

## Results and discussion

### Chemistry

**Scheme 1.**
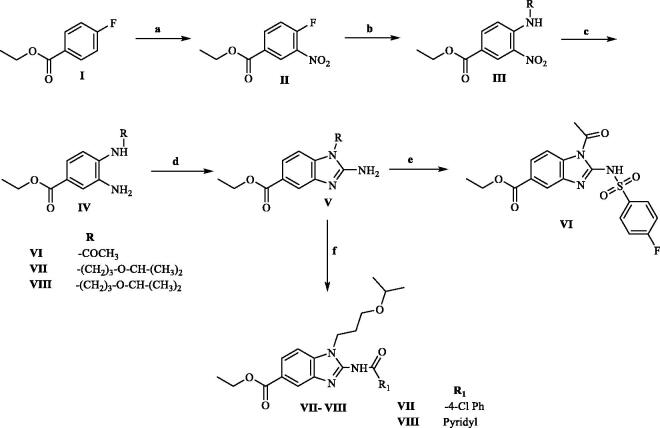
Reagents and conditions (**a**) H_2_SO_4_, NaNO_3_, r.t, 2 h, (**b**) RNH_2_, K_2_CO_3_, DMF, 70 °C, overnight, (**c**) Pd/ H_2_, CH_3_OH, N_2_, r.t, overnight, (**d**) CNBr, NaHCO_3_, CH_3_OH, r.t, 2 h, (**e**) 4-Fluorobenzenesulfonyl chloride, pyridine, N_2_, r.t, overnight, (**f**) RCOOH, TBTU, DMAP, N_2_, DMF, r.t, overnight.

**Scheme 2.**
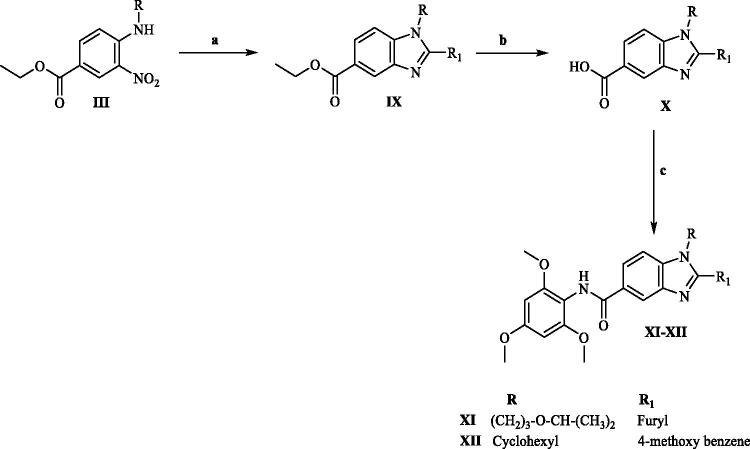
Reagents and conditions (**a**) RCHO, Na_2_S_2_O_4_, DMSO, 120 °C, 7 h, (**b**) LiOH, ethanol, reflux, 2 h, (**c**) RNH_2_,TBTU, DMAP, N_2_, DMF, r.t, overnight.

The synthetic pathways for the preparation of our target compounds were outlined in Schemes 1 and 2. The newly synthesised compounds were characterised by their melting points, ^1^H NMR, ^13^C NMR and LC/MS spectra, in which ^1^H NMR signals were consistent with protons of the synthesised target compounds (**VI, VII**, **VIII, XI** and **XII**). The synthesis of 1,2,5-substituted benzimidazole derivatives was started with the nitration of ethyl 4-fluorobenzoate (**I**)^27^ using a mixture of concentrated sulphuric acid and NaNO_3_ according to the reported procedure^28^ followed by amination of ethyl 3-nitro-4-fluorobenzoate (**II**) *via* nucleophilic aromatic substitution using K_2_CO_3_ as a base^29^ and dimethylformamide (DMF) as a polar aprotic solvent to yield ethyl 3-nitro-4-(amino)benzoate derivatives (**III**). Cyclisation of 2-amino substituted benzimidazole ring was achieved by the catalytic reduction of compound (**III**) derivatives using H_2_/Pd^30^ to yield the reduced (**IV**) derivatives which were further reacted with cyanogen bromide according to the reported procedure^31^ to yield 2-amino benzimidazole derivatives (**V**). The final compound (**VI**) was obtained by the reaction of compound (**V**) with 4-fluorobenzenesulfonyl chloride in pyridine as acid scavenger under N_2_ atmosphere^32^. ^1^H NMR signals of compound (**VI**) showed the appearance of doublet signals at δ 7.97 and 7.38 corresponding to the protons of 4-fluorobenzenesulfonyl moiety. On the other hand, the final compounds (**VII**) and (**VIII**) were obtained *via* direct coupling and amide bond formation through the reaction of (**V**) with 4-chlorobenzoic acid and isonicotinic acid, respectively, using tetramethyl benzotriazole uronium tetrafluoroborate (TBTU) in the presence of 4-dimethylaminopyridine (DMAP) under N_2_ atmosphere^33^. The ^1^H NMR signals of compounds (**VII**) and (**VIII**) revealed aliphatic signals of the isopropoxy propyl moiety. Additionally, aromatic protons of 4-chlorobenzene moiety were significant in ^1^H NMR chart of compound (**VII**) at δ 8.21 and 7.61 whereas protons of pyridyl moiety in compound (**VIII**) appeared as doublets at δ 8.75 and 8.10. The final compounds (**XI)** and (**XII**) were obtained by one pot reaction and reductive cyclisation of *N*-alkyl ethyl 3-nitrobenzoate (**III**) with the furan-2-aldehyde and 4-methoxy benzaldehyde, respectively, in the presence of sodium dithionite as a reducing agent according to the reported procedure^34^ to yield (**IX**) derivatives which were further subjected to ester hydrolysis by refluxing with LiOH in the presence of ethanol/water^35^^,^^36^ to yield the corresponding acid derivatives. The acid products were reacted with the 2,4,6 trimethoxyaniline in dry DMF in the presence of DMAP and TBTU^33^ to yield the final compounds (**XI**) and (**XII**). ^1^H NMR signals of compounds (**XI**) and (**XII**) revealed the disappearance of the triplet and doublet ester signals at δ 2 and 1.35, respectively. Moreover, new singlet signal appeared at δ 3.37 resulting from the 9 protons of 2,4,6-trimethoxybenzene moiety. Compound (**XI**) was confirmed as two exclusive signals of the furyl group were apparent at δ7.31 and 7.15 in the ^1^H NMR chart. Finally, compound (**XII**) was confirmed as cyclohexyl group revealed ^1^H NMR signals at δ 2.31–1.28 with their respective integration.

### Biological evaluation

Doxorubicin (Dox) is one of the well-established anticancer drugs used in the treatment of different types of cancer, including leukaemia, breast, lung, gastric and paediatric cancers^37^. Additionally, it is commonly used as reference cytotoxic agent in different biological evaluation tests, especially cytotoxicity assays. However, cancer cells which are treated with doxorubicin acquire multidrug resistance after some time of treatment^38^. Acute and/or delayed toxicities or even both, have been reported in patients receiving doxorubicin in addition to dangerous side effects including bone marrow depression, cardiomyopathy and sever diarrhea^39^. Therefore, one of the main concerns should be the use of lower doses of doxorubicin to decrease such destructive side effects.

In order to evaluate the cytotoxic activity of our synthesised compounds, initial screening tests were performed for our synthesised compounds in doxorubicin sensitive cell lines CCRF/CEM (leukaemia) and MCF7 (breast cancer). Afterwards, the IC_50_ values of the promising antiproliferative candidates were evaluated in doxorubicin sensitive cells and doxorubicin resistant cells CEM/ADR500 (leukaemia) and Caco-2 (colon cancer) as well. This was followed by testing the ABCB1 inhibitory activities of the most promising cytotoxic compounds through rhodamine 123 (Rho123) assay in doxorubicin resistant cells CEM/ADR500 where ABCB1 transporters are overexpressed. The ABCB1 inhibitory effects of our promising compounds were ascertained when used in combinations at non-toxic doses (IC_10_, IC_20_ and IC_30_) with doxorubicin in doxorubicin resistant cells CEM/ADR500 and Caco-2 and the effects of such combinations on IC_50_ of doxorubicin were recorded and evaluated.

#### Cytotoxicity (MTT assay)

**Figure 5.**
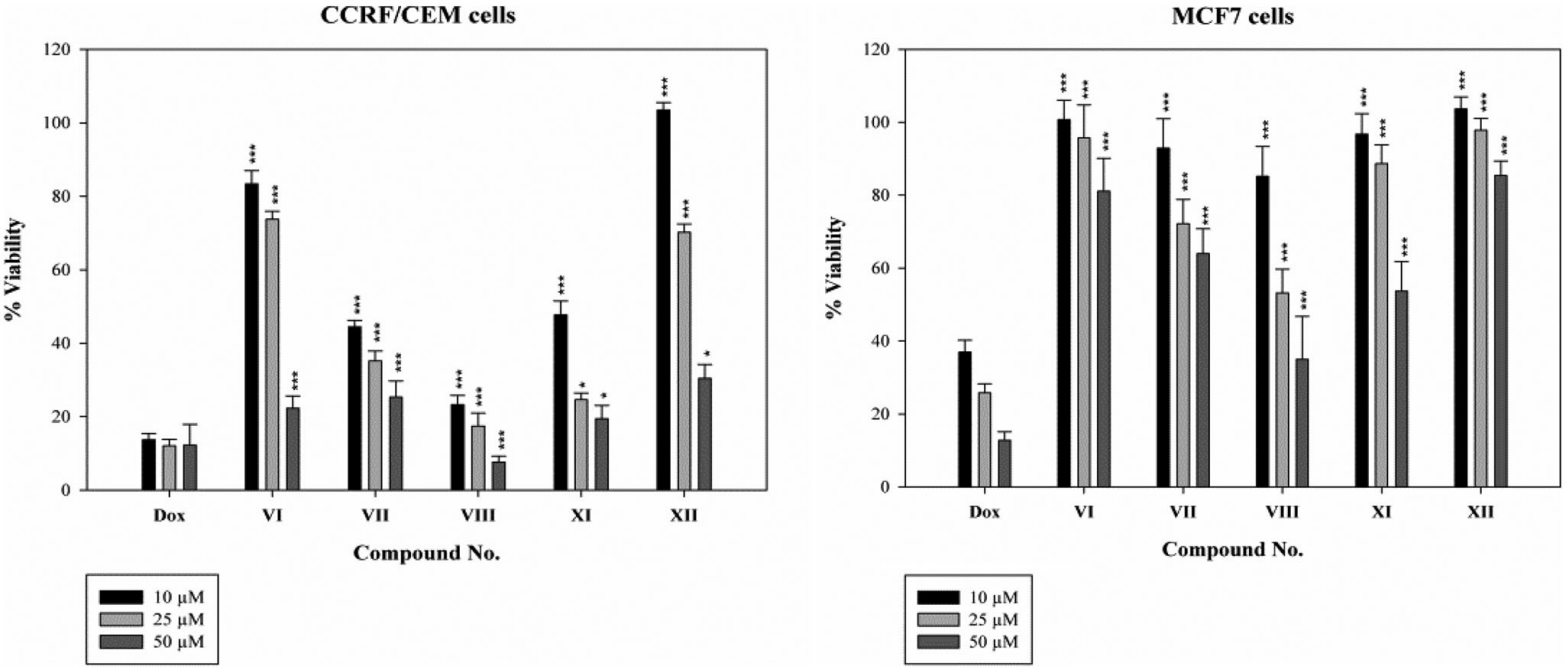
Results of screening test for the cytotoxic effects of target benzimidazole compounds against doxorubicin sensitive cell lines (CCRF/CEM and MCF7) at three concentrations (10, 25 and 50 µM). Data represent the mean ± standard deviation of the mean of three independent experiments. All results are significantly different from doxorubicin using analysis of variance (One-Way Anova), where; *p ˂ 0.05, ***p ˂ 0.001.

Our synthesised benzimidazole based compounds were preliminary screened for their inhibitory action in doxorubicin sensitive cell lines (CCRF/CEM and MCF7) at three dose levels (10, 25 and 50 µM) (Figure 5). Afterwards, the IC_50_ values of the cytotoxic target compounds were evaluated in the doxorubicin sensitive cells (CCRF/CEM and MCF7) as well as doxorubicin resistant cells (CEM/ADR5000 and Caco-2) *via* MTT assay using doxorubicin (Dox) as reference compound (Table 1). The close investigation of the structure activity relationship of the targeted compounds reflected that the 2-amido-5-carboxylate derivatives (**VII** and **VIII**) exhibited substantial cytotoxic activities in CCRF/CEM, CEM/ADR5000 and Caco-2 cells as showed by the results of the MTT assay in the tested cell lines. Remarkably, the 1-isopropoxypropyl-5-amido derivative (**XI**) revealed well tolerated cytotoxic effects, whereas the 1-cyclohexyl-5-amido derivative (**XII**) showed no cytotoxic activity as well as the 2-sulfoamido-5-carboxylate derivative (**VI**). Collectively, the presence of an acyclic aliphatic substitution at the 1-position of the benzimidazole ring together with an amido group at the 2-position would increase the cytotoxic activity of the benzimidazole derivative. The results of the MTT assay reflected the substantial cytotoxic activities of the 5-carboxylate substituted compounds (**VII**- **VIII**) and the 5-amido substituted compound (**XI)** in CCRF/CEM cell line (IC_50_ = 6.63 µM, 3.61 µM and 4.45 µM, respectively) compared to that of doxorubicin (IC_50 =_ 0.20 µM). Thus, the cytotoxic effect was retained with bioisoteric replacement at such position. Whereas in MCF7 cells, only compound (**VIII**) which bears a pyridyl group showed some cell inhibition (IC_50_ = 23.30 µM) in comparison to doxorubicin (IC_50_ = 6.91 µM). Interestingly, compounds (**VII**), (**VIII**) and (**XI**) showed high cytotoxic activity in CEM/ADR 5000 resistant leukaemia cells (IC_50_ = 22.02 µM, 8.13 µM and 11.20 µM, respectively) compared to doxorubicin (IC_50_ = 34.50 µM). Moreover, in Caco-2 resistant cell lines, compounds (**VII**) and (**VIII**) showed comparable inhibition (IC_50_ = 13.47 µM and 13.86 µM, respectively) with respect to that of doxorubicin (IC_50_ =11.69 µM). The previous results demonstrate that among our synthesised compounds, compound (**VIII**) had the best cytotoxic activity in all tested cell lines, revealing that 1-alkyl, 2-amido, 5-carboxylate benzimidazole derivatives are promising candidates for the design of cytotoxic compounds.

**Table 1.** IC_50_ values in (µM) for cytotoxic target compounds in different cell lines. Doxorubicin (Dox) was used as positive control.

Compound	CCRF/CEM	MCF7	CEM/ ADR 5000	Caco-2
**Dox**	0.20 ± 0.01	6.91 ± 0.17	34.50 ± 0.67	11.69 ± 0.32
**VII**	6.63 ± 0.21	ND	22.02 ± 0.79	13.47 ± 0.34
**VIII**	3.61 ± 0.11	23.30 ± 0.68	8.13 ± 0.24	13.86 ± 0.27
**XI**	4.45 ± 0.07	ND	11.20 ± 0.16	ND

Data are represented as mean ± standard derivation. Each IC_50_ value was determined in three independent experiments. ND means >50 µM.

#### ABC transporter activity

**Figure 6.**
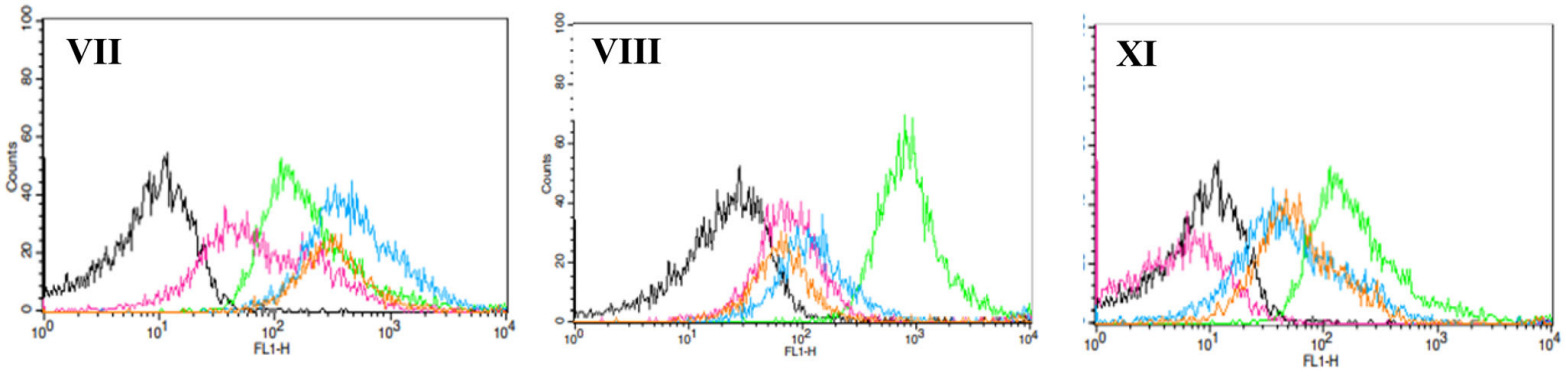
Histograms of flow cytometry of Rho 123 retention in CEM/ADR 5000 cells after 1.5 h treatment with three concentrations (IC_50_ (pink), 50 µM (blue) and 100 µM (orange)) of a compounds **VII**, **VIII** and **XI** compared to the treatment with 50 µM verapamil (positive control, green). All experiments were repeated three times.

The human leukaemia doxorubicin-resistant cell line CEM/ADR 5000 is well known for its overexpression of ABCB1 transporters^40^. Our cytotoxic benzimidazole target compounds (**VII**), (**VIII**) and (**XI**) were tested for their possible modulation of ABCB1 activity in CEM/ADR5000 cells, and hence, their multidrug resistance inhibition. This was assessed *via* rhodamine 123 flow cytometric assay, using verapamil (**1**) as a reference compound. This assay aimed to evaluate the ability of the tested compounds to decrease the cytotoxic drug efflux by ABCB1 transporters. Rhodamine 123 (Rho123) is accumulated intracellularly when the ABCB1 transporters are inhibited. The effects of the tested compounds on ABCB1 activity were evaluated in CEM/ADR5000 leukaemia cells at three different concentrations (IC_50_, 50 µM and 100 µM) as demonstrated in Figure 6. The results of the flow cytometric assay presented below show that treatment of CEM/ADR 5000 cells with 2-amido p-chlorophenyl, 5-carboxylate derivative (**VII**) resulted in significant accumulation of Rho123 inside the cells at all previously mentioned concentrations compared to DMSO control (<0.1%). Interestingly, the Rho123 accumulation observed in cells treated compound (**VII**) at doses (50 µM and 100 µM) is comparable to that observed in cells treated with verapamil, thus ABCB1 activity is affected. Treatment of cells with 2-amido pyridyl compound (**VIII**) showed some accumulation of Rho123 inside cells higher than that observed in cells treated with DMSO control, but less than accumulation in cells treated with verapamil at the three used concentrations. Unfortunately, the 5-amido substituted target compound (**XI**) showed some Rho123 intracellular retention at higher concentration (100 µM) but same as DMSO control when used in concentration IC_50_. From the previous results we can conclude that 2-amido, 5-carboxylate benzimidazole derivatives are favourable for the design and development of ABCB1 inhibitors.

#### The effect of combination treatments in doxorubicin resistant cells

**Figure 7.**
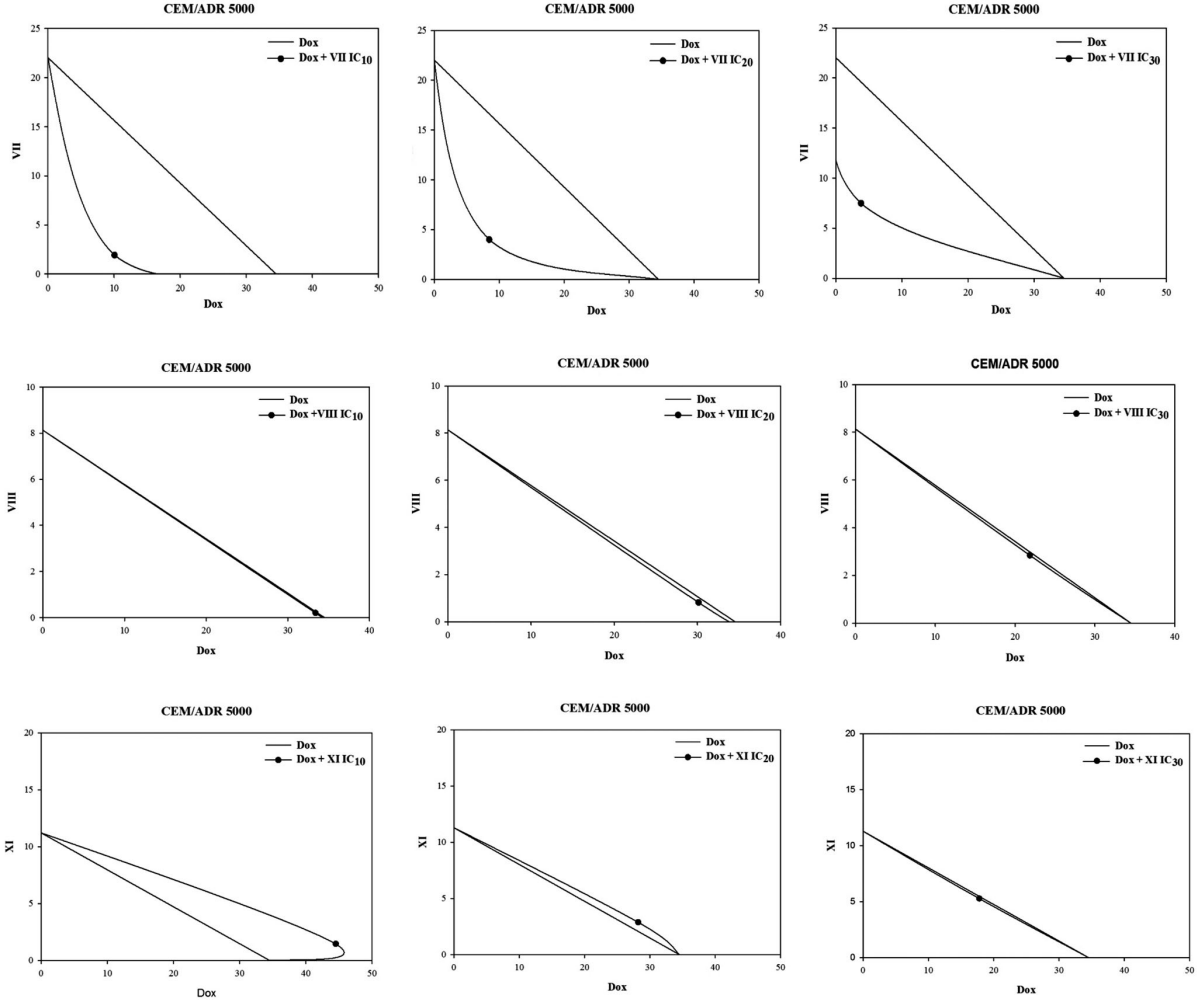
Isobologram analysis of the effects of combinations treatments in CEM/ADR 5000 cells; the IC_50_ of doxorubicin and the used concentrations of the target benzimidazole derivatives are plotted on the x-axis and y-axis, respectively. The line connecting these two points is the line of additivity. The point in the picture corresponds to the IC_50_ of Dox obtained after combination (x-axes) and the fixed concentration of the target compound used in the combination (y-axes). Points located below the line indicate synergy. Dox: doxorubicin.

**Figure 8.**
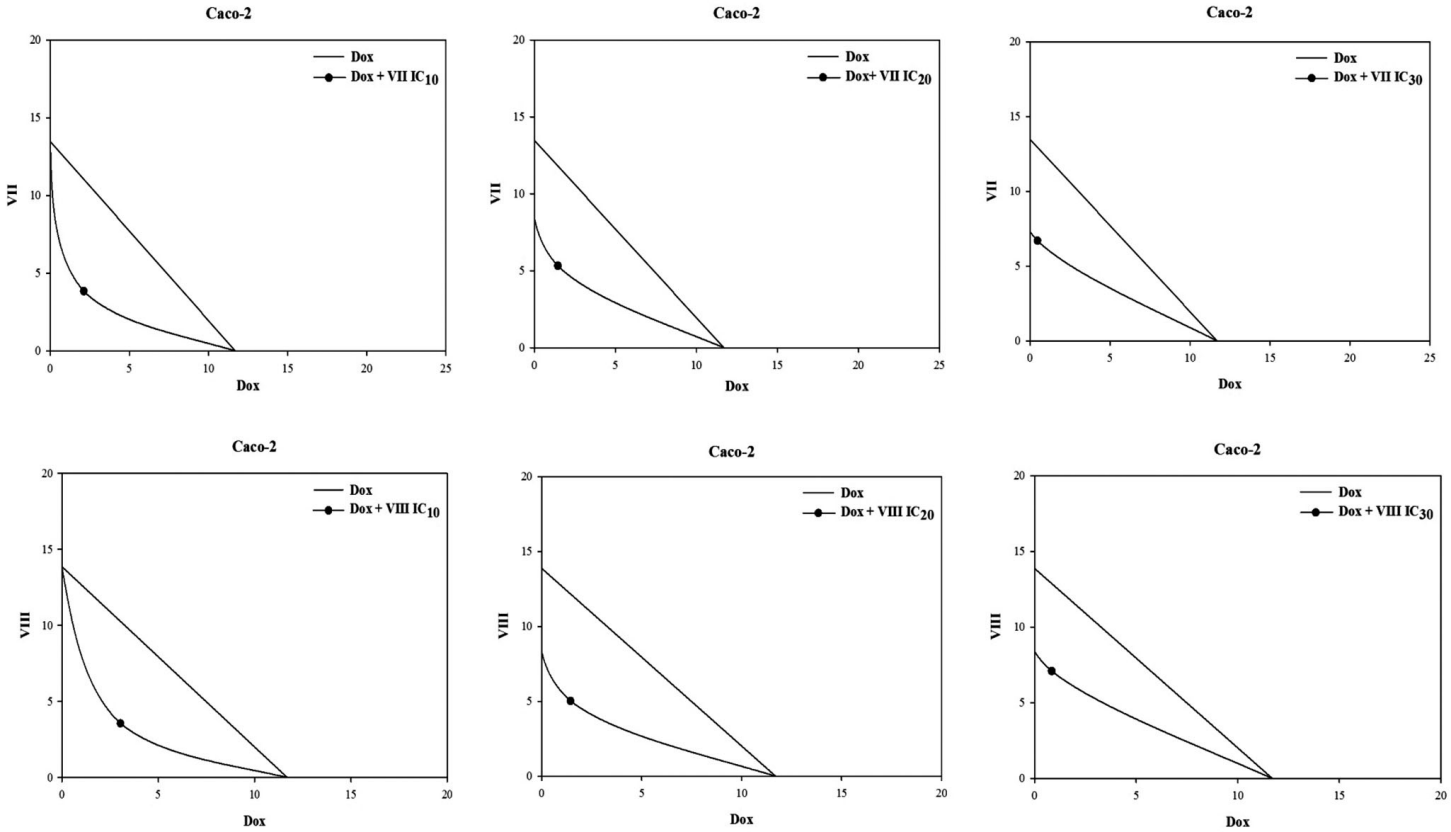
Isobologram analysis of the effects of combinations treatments in Caco-2 cells; the IC_50_ of doxorubicin and the used concentrations of the target benzimidazole derivatives are plotted on the x-axis and y-axis, respectively. The line connecting these two points is the line of additivity. The point in the picture corresponds to the IC_50_ of Dox obtained after combination (x-axes) and the fixed concentration of the target compound used in the combination (y-axes). Points located below the line indicate synergy. Dox: doxorubicin.

The combinations of doxorubicin together with fixed non-toxic concentrations (IC_10_, IC_20_ and IC_30_) of our most potent cytotoxic compounds (**VII**), (**VIII**) and (**XI**) were tested in CEM/ADR5000 and Caco-2 resistant cell lines and the IC_50_ values of doxorubicin were recorded. Regarding CEM/ADR5000 cells, synergistic effects were observed in all combination treatments of doxorubicin and compound (**VII**) (IC_10_, IC_20_ and IC_30_). Noteworthy, compound (**VII**) also showed marked ABCB1 inhibition in Rho 123 assay.However, an additive effect was observed in the combination treatments using compound (**VIII**) (IC_10_, IC_20_ and IC_30_). Unfortunately, antagonistic effects were apparent in combinations of the 5-amido benzimidazole derivative (**XI**) **(**IC_10_ and IC_20_) and doxorubicin but some additive effect was noticed at concentration of IC_30_ (Figure 7, Table 2). Notably, compound **(XI)** failed to inhibit ABCB1 transporters at concentration below 100 µM in Rho 123 assay. Interestingly, synergistic effects were recorded in combinations of doxorubicin and the 2-amido, 5-carboxylate benzimidazole compounds (**VII**) and (**VIII**) (IC_10_, IC_20_ and IC_30_) in Caco-2 cell lines (Figure 8, Table 3). Synergistic effects and additive effects observed in the previously mentioned combination treatments are supposed to be as a result of the ability of compounds (**VII**) and (**VIII**) to modulate ABCB1 transporters, hence decrease doxorubicin efflux and increase its intracellular concentration. The mean of the combination index (CI) from data points was calculated according to the Chou-Talalay method^41^.

**Table 2.** IC_50_ values in (µM) for doxorubicin in combination with IC_10_, IC_20_ and IC_30_ of compounds (**VII**, **VIII** and **XI**) in CEM/ADR 5000 resistant cell line.

		CEM/ADR 5000 IC_50_ μM of Dox	CI	IB	Interpretation
**Doxorubicin alone**		34.50 ± 0.67	NR	NR	
**Dox + VII**	**IC_10_**	10.06*** ± 0.10	0.38	syn.	(+++)
	**IC_20_**	8.46*** ± 0.11	0.43	syn.	(+++)
	**IC_30_**	3.81*** ± 0.10	0.46	syn.	(+++)
**Dox + VIII**	**IC_10_**	33.41* ± 0.81	1.10	add.	(±)
	**IC_20_**	31.87*** ± 0.33	1.10	add.	(±)
	**IC_30_**	21.85*** ± 0.43	1.03	add.	(±)
**Dox + XI**	**IC_10_**	44.53*** ± 0.83	1.42	ant.	(--)
	**IC_20_**	28.21*** ± 0.57	1.17	ant.	(-)
	**IC_30_**	17.79*** ± 0.54	1.06	add.	(±)

Data are represented as mean ± standard derivation. Each IC_50_ value was determined in three independent experiments. All results are significantly different from doxorubicin using student paired t-test, where; *p ˂ 0.05, ***p ˂ 0.001.

**Table 3:** IC_50_ values in (µM) for doxorubicin in combination with IC_10_, IC_20_ and IC_30_ of compounds (**VII** and **VIII**) in Caco-2 resistant cell lines. Data are represented as mean ± standard derivation.

		Caco-2 IC_50_ μM of Dox	CI	IB	Interpretation
**Doxorubicin**		11.69 ± 0.32	NR	NR	
**Dox + VII**	**IC_10_**	2.12*** ± 0.04	0.47	syn.	(+++)
	**IC_20_**	1.45*** ± 0.11	0.52	syn.	(+++)
	**IC_30_**	0.46*** ± 0.02	0.54	syn.	(+++)
**Dox + VIII**	**IC_10_**	3.04*** ± 0.13	0.52	syn.	(+++)
	**IC_20_**	1.44*** ± 0.10	0.48	syn.	(+++)
	**IC_30_**	0.84*** ± 0.07	0.58	syn.	(+++)

Each IC_50_ value was determined in three independent experiments. All results are significantly different from doxorubicin using student paired t-test, where; *** *p* ˂ 0.001.

#### Overview on combination index (CI) for combination effects

This shows CI for each one of the tested compounds added in combination with doxorubicin for the tested cell lines. All CI values are rated: <0.1, very strong synergism (+++++); 0.1 to 0.3 strong synergism (++++); 0.3 to 0.7, moderately strong synergism (+++); 0.7 to 0.85, moderate synergism (++); 0.85 to 0.9, slight synergism (+); 0.9 to 1.1, nearly additive synergistic effect (±); 1.1 to 1.2 slight antagonism (-); 1.2 to 1.45, moderate antagonism (–); 1.45 to 3.3, moderately strong antagonism (–-); 3.3 to 10, strong antagonism (––); >10, very strong antagonism (––-)^41^. Interpretation of the isobolograms (IB) is abbreviated as follows: add: additive effect; ant: antagonism; syn: synergism. NR = not relevant.

### In silico studies

#### Ligand based pharmacophore design and ligand pharmacophore mapping

The ligand-based pharmacophoric model built after the molecular hybridisation of reference compounds WS-*691* (**3**) and BZD9L1 (**12**) was achieved using Accelrys Discovery Studio® 2.5.5 software. Reported data revealed that the benzimidazole ring and 4-fluoroaniline in WS-*691* (**3**) are involved in binding with key amino acids in the binding site of ABCB1 through pi-pi interactions, whereas the fluorine atom was involved in hydrogen bonding. On the other hand, the reported ligand interactions of the anticancer agent BZD9L1 (**12**) demonstrated that the N atom from the imidazole moiety and the ester group at the 5-position of benzimidazole ring were involved in hydrogen bonding which is responsible for its anticancer activity. Other prominent features included hydrophobic interactions and ring aromatic feature. Mapping of the proposed compounds over the hybridised pharmacophore was performed using ligand pharmacophore mapping protocol, Accelrys Discovery Studio® 2.5.5 software. The results of mapping of our designed compounds revealed that the mapped compounds showed well fitting in the aromatic ring features in both ABCB1 inhibitor WS-*691* (**3**) and BZD9L1 (**12**) except the 2-sulfonamide derivative (**VI)**. Results shown in Table 4 also demonstrated the best fitting values with compounds (**VII** and **VIII**) as the 5-carboxylate moiety well fit with the hydrophobic feature in the pharmacophore of the anticancer BZD9L1 (**12**). However, the 5-amido derivatives (**XI** and **XII**) showed less fitting with such hydrophobe. Moreover, compound (**XII**) bearing the 1-cyclohexyl substitution missed the H-bond acceptor feature in ABCB1 pharmacophore which decreased its FitValue than compound (**XI**). Unfortunately, Compound (**VI**) showed the least FitValue as the groups are not well fitted to the centre of the pharmacophoric features. According to the previously mentioned results, we were encouraged to synthesise our proposed 1,2,5-trisubstituted benzimidazole derivatives and evaluate their biological activity as cytotoxic candidates able to modulate ABCB1 to overcome multidrug resistance especially in resistant cancer cells.

**Table 4:** FitValues after ligand pharmacophore mapping of designed target compounds.

Compound	VI	VII	VIII	XI	XII
**FitValue**	5.66	6.51	6.78	6.24	5.92

#### Molecular modelling

*In silico* studies were performed to investigate the binding modes of the synthesised benzimidazole compounds at the ABCB1 binding site in comparison to the reported interactions of ABCB1 inhibitor zosuquidar (**2**) and the docked pose of WS-*691* (**3**) inhibitor as well.

**Figure 9.**
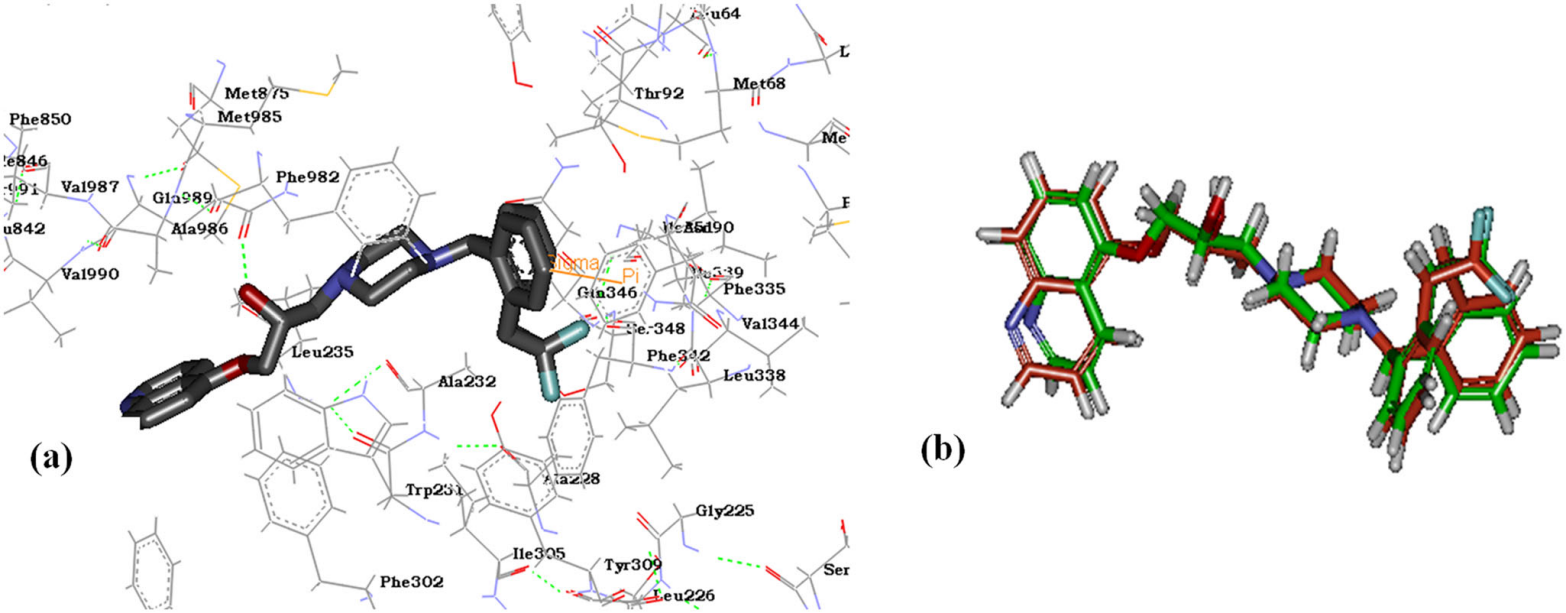
(**a**) 3 D diagram showing the binding interactions of reference compound zosuquidar (**2**) at ABCB1 active site, (**b**) Alignment of the original pdb pose of zosuquidar (Green) and the docked pose for RMSD value calculation.

**Figure 10.**
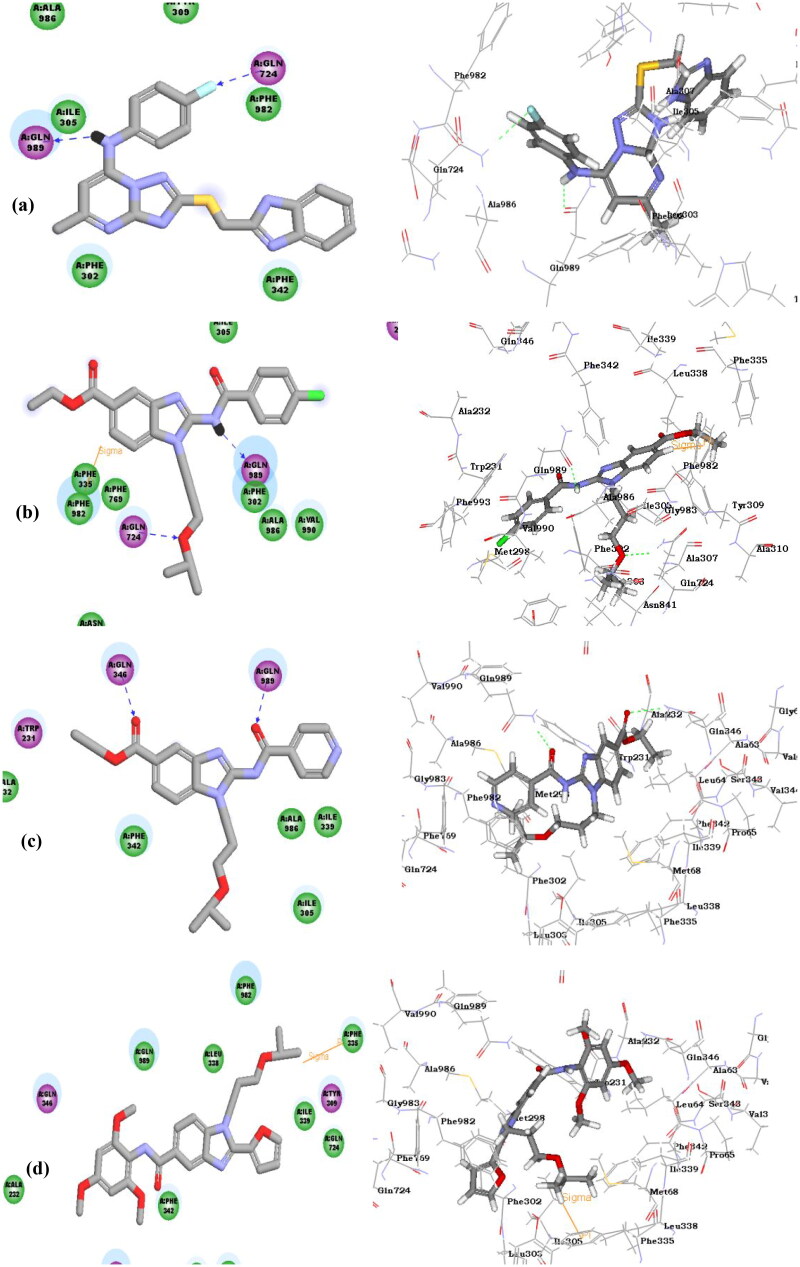
**(a–d)** Docked poses of the target compounds WS-*691* (**3**), (**VII**), (**VIII**) and (**XI**) at ABCB1 active site (code: 6QEE) in 2 D and 3 D representations. (**a**) Docked pose of WS-*691* (**3**); (**b**) Docked pose of (**VII**); (**c**) Docked pose of (**VIII**); (**d**) Docked pose of (**XI**).

A docking study was performed using CDOCKER protocol, Accelrys Discovery Studio® 2.5.5 software. The results obtained aided to get an overview about the structure activity relationship of the synthesised benzimidazole target compounds and the important groups for interaction. Also, the docking results served to better understand the ABCB1 inhibition observed in resistant CEM/ADR 5000 cells. The docking results of our target compounds were compared to the reported interactions of the ABCB1 inhibitor zosuquidar (**2**) with key aminoacids at ABCB1 active site. The X-ray crystal structure of ABCB1 co-crystallized with the ABCB1inhibitor zosuquidar (**2**) was obtained from protein data bank (PDB: **6QEE**). The main key interactions observed with the zosuquidar molecule is the hydrogen bonding with Gln 989 together with pi-sigma bond with Phe 335 (Figure 9(a)). Such interactions may explain the wide range and diversity of ABCB1 transporters substrates, and may give an idea about the lack of specificity of ABCB1 inhibitors evolved. The RMSD value was calculated for the original pdb pose of zosuquidar and the docked pose in order to validate the CDOCKER protocol used for docking, and confirm its predictability of the correct poses. The calculated RMSD value of 0.57°A was obtained (Figure 9(b)) supporting the used CDOCKER protocol to anticipate ligand-protein interactions in the X-ray structure of ABCB1. The docked pose of WS-*691* (**3**) together with the docked poses of the cytotoxic benzimidazole target compounds as ABCB1 inhibitors (**VII**, **VIII** and **XI**) were compared to the original pose of the zosuquidar inhibitor (**2**) obtained from pdb. The binding modes and interactions with amino acid moieties at the active site are shown below (Figure 10(a–d)) and CDOCKER interaction energies are listed in Table 5. The ABCB1 inhibitor WS-*691* (**3**) showed hydrogen bonding with the key aminoacid Gln 989 in addition to hydrogen bond with Gln 724. Interestingly, target compound (**VII**) which exhibited the best ABCB1 inhibition in Rho123 assay showed a comparable binding mode and CDOCKER interaction energy to those observed in reference compounds zosuquidar (**2**) and WS-*691* (**3**).

**Table 5:** CDOCKER interaction energies using CDOCKER protocol.

Compound	CDOCKER Interaction Energy
**Zosuquidar (2)**	39.76
**WS-*691* (3)**	39.01
**VII**	38.75
**VIII**	37.38
**XI**	35.26

The docking results of target compound (**VII**) at the ABCB1 active site demonstrated two key hydrogen bonds, the NH-atom of the 2-amido group with Glu 989 and the oxygen atom in the isopropoxy propyl moiety with Gln 724 in addition to sigma-pi interaction with Phe 982. Moreover, the target compound (**VIII)** showed hydrogen bonding of the carbonyl group of the 2-amido substitution with Gln 989 and another H-bond the carbonyl group of the ester moiety with Gln 346. However, the docking pose of compound (**XI**) bearing the 2-furyl group directly attached at the 2-position and an amide substitution at the 5-position showed sigma- pi interaction with Phe 335 only. The binding modes and the CDOCKER interactions energies are well correlated to the results obtained in the Rho123 assay for testing ABCB1 inhibitory activity.

#### In vitro AMDE study of the physiochemical properties of target compounds using lipiniski rule

To get a deeper insight about the physiochemical properties of our cytotoxic target compounds (**VII**), (**VIII**) and (**XI**), Lipiniski rule protocol, Molecular operating environment (MOE) software. Lipiniski rule of five gives a description if a biologically active molecule could exhibit an oral bioavailability based on its chemical and physical properties. The ADME properties (absorption, distribution, metabolism and excretion) are based upon specific molecular properties as hydrogen bond acceptor not more than 10, hydrogen bond donor not more than 5, LogP not more than 5 and molecular weight not more than 500 Da. A molecule is considered an orally bioavailable if it fulfils 2 or more of the previously mentioned conditions^42^^,^^43^. Results showed that our cytotoxic target compounds (**VII**), (**VIII**) and (**XI**) could exhibit an oral bioavailability. Interestingly, compound (**VIII**) fulfilled all the conditions of Lipiniski rule, whereas compounds (**VII)** and (**XI)** skipped only one condition but still show the possibility to exhibit oral bioavailability (Table 6). These findings suggest that 5-carboxylate moiety is better for oral absorption than amido group at the same position as ester group is more easily absorbed and metabolised than amide group. Finally, the presence of the pyridyl group in compound (**VIII**) was better for the oral bioavailabilty than p-chloro benzene ring in compound (**VII**). This maybe attributed to the basic property and considerable water solubility of pyridine ring.

**Table 6:** *In vitro* ADME study for cytotoxic benzimidazole target compounds using Lipiniski rule of five.

Compound	H.acceptor	H.donor	Drugability.	LogP (o/w)	M.wt	Lip.violation
**VII**	7.00	1.00	1.00	5.07	443.93	1
**VIII**	8.00	1.00	1.00	3.24	410.47	0
**XI**	9.00	1.00	1.00	4.47	493.56	1

## Conclusion

Novel 1,2,5-trisubstituted benzimidazole derivatives were designed, synthesized and evaluated for their cytotoxic effects in doxorubicin sensitive cell lines (CCRF/CEM and MCF7) and doxorubicin resistant cell lines (CEM/ADR 5000 and Caco-2). Target compounds **VII**, **VIII** and **XI** showed significant cytotoxic effects in the CCRF/CEM, CEM/ADR 5000 and Caco-2 cell lines but no significant activity in MCF7 cells except for compound **VIII** which showed some cell inhibition in the aforementioned cell line. Furthermore, the target compound **VII** showed the best ABCB1 inhibitory action at all tested concentrations (IC_50_, 50 µM and 100 µM) in rhodamine 123 flowcytometric assay. Remarkably, compound **VII** showed favourable synergetic effects when tested in combination at non-toxic doses with doxorubicin in doxorubicin resistant cells. These synergistic effects are supposed to come up as a result to ABCB1 inhibition by compound **VII**, leading to the decrease in doxorubicin efflux and the increase in its intracellular concentration. The *in silico* studies also showed the well-fitting of compound **VII** at the ABCB1 binding site and its interaction with the key aminoacid Gln 989 with additional hydrogen bonding with Gln 724 and pi-sigma interaction with Phe 982. Moreover, compound **VII** fulfilled the Lipiniski rule of five supporting its predictive oral bioavailability. Consequently, 1,2,5-trisubstituted benzimidazole derivatives are promising candidates for optimisation and development of anticancer agents with ABCB1 inhibition to overcome MDR.

## Experimental

### Chemistry and analysis

Starting materials and reagents were purchased from Sigma-Aldrich (USA, Germany), and Alfa Aesar (Germany) and were used without further purification. Different solvents were purchased from Sigma-Aldrich and Fisher scientific and used directly without further purification. Solvents for column chromatography as ethyl acetate and hexane were dried by distillation. Reactions were followed up using analytical thin layer chromatography (TLC), on silica gel 60 F_254_ packed on aluminium sheets (Merck®), and were visualised under UV light (254 nm). Intermediates monitoring was carried out using melting points. **^1^H NMR** spectra were determined on a Varian NMR instrument at **300 MHz** in δ scale (ppm) and J (Hz) and referred to the deuterated solvent peak (DMSO-d_6_ δ = 2.5 ppm). **^13^C NMR** spectra were determined on the same instrument at **75 MHz** and referred to the solvent peak (DMSO-d_6_ δ = 39.52 ppm). LC-MS/MS analysis was performed on a Finnigan LCQ-Duo ion trap mass spectrometer with an ESI source (ThermoQuest) coupled to a Thermo Scientific Accela HPLC system (MS pump plus, autosampler, and PDA detector plus). Data acquisitions and analyses in LC-MS/MS were executed by Xcalibur^TM^ 2.0.7 software (Thermo Scientific). Compound (**I**) was purchased from Sigma-Aldrich (USA) and used as such without purification.

#### Ethyl 4-fluoro-3-nitrobenzoate (II)

The starting material ethyl 4- fluorobenzoate (16.817 g; 100 mmol) was added portionwise onto concentrated H_2_SO_4_ (150 ml) at 0 °C. The mixture was frozen for 2 h before the addition of NaNO_3_ (9.35 gm, 110 mmol) which was added portionwise over 1 h in an ice bath with continuous stirring. The mixture was allowed to stir for 1 h in ice and 1 more hour at room temperature before being poured over ice and washed well with ice cold water and extracted with ethylacetate (150 ml). The organic layers were combined, washed with saturated NaHCO_3_ solution (100 ml), dried over anhydrous Na_2_SO_4_ and concentrated under reduced pressure to give ethyl 4-fluoro-3-nitrobenzoate (**II**) as a pale yellow solid with a yield of (10.77 g, 95%); m.p. 42–44 °C as reported^27^^,^^28^.

#### Ethyl 3-(alkylamino)-4-nitrobenzoate (III)

*General procedure:* The appropriate amine (1.1 equiv; 11 mmol) was added to a stirred solution of ethyl 4-fluoro-3-nitrobenzoate (**II**) (2.14 g, 10 mmol) and K_2_CO_3_ (1.66 g, 20 mmol) in 20 ml DMF. After overnight reflux at 70 °C, the mixture was poured onto ice water, stirred for 10 min, filtered and dried to give ethyl 3-(alkylamino)-4-nitrobenzoate (**III**) with a yield of (85–90%).

#### Ethyl 3-(alkylamino)-4-aminobenzoate (IV)

*General procedure:* To the stirred solution of ethyl 3-(alkylamino)-4-nitrobenzoate (**III**) in methanol, H_2_/Pd was added (10%) portionwise and allowed to stir overnight at room temperature under H_2_ atmosphere. The reaction was followed up by TLC. After reaction completion, the mixture was filtered over celite then concentrated and used directly in the next reaction (yield 80%).

#### Ethyl 2-amino-1-alkyl-1H-benzo[d]imidazole-5-carboxylate (V)

*General procedure:* to the stirred cooled solution of the respective ethyl 3-(alkylamino)-4-amino-benzoate (**IV**) (1 equiv, 40 mmol) in methanol (30 ml), cold suspension of cyanogen bromide (5 g, 1.25 equiv, 50 mmol) in water (30 ml) was added over a period of 5 min with rapid stirring. The reaction mixture was stirred for 45 min at room temperature, solid sodium bicarbonate (3.36 g, 40 mmol) was added in small portions over a period of 1.5 h to bring the pH to 6.5 − 7.0. Stirring was continued for another 1 h. The solid was separated by filtration, washed with cold water (3*20 ml) to yield benzimidazole derivatives (**V**) with yield of (70–80%).

#### Ethyl-1-acetyl-2-((4-fluorophenyl)sulfonamido)-1H-benzo[d]imidazole-5-carboxylate (VI)

To a solution of ethyl 1-acetyl-2-amino-1H-benzo[d]imidazole-5-carboxylate (**V**) (4.95 g, 20 mmol) in dry pyridine (10 ml), 4-fluorobenzenesulfonyl chloride (5.83 g, 30 mmol) was added dropwise. The solution turned yellow upon addition. The reaction was allowed to stir overnight at room temperature under N_2_ atmosphere and followed by TLC. Upon completion, the solution was evaporated, washed with ice cold water and dried to afford the titled compound (**VI**). White solid, yield 60%; m.p. 188–190 °C; **^1^H NMR (300 MHz, DMSO-d_6_)** δ 12.08 (s, 1H), 8.06 (s, 1H), 7.97 (dd, *J* = 8.9, 5.3 Hz, 2H), 7.80 (dd, *J* = 7.8, 1.5 Hz, 1H), 7.68 (d, *J* = 1.3 Hz, 1H), 7.38 (d, *J* = 8.8 Hz, 2H), 4.31 (qd, *J* = 7.1, 0.9 Hz, 2H), 2.50 (s, 3H), 1.32 (t, *J* = 7.1 Hz, 3H).**^13^C NMR (75 MHz, DMSO-d_6_)** δ 165.8, 165.6, 149.4, 140.4, 132.4, 132.34, 129.8, 129.0, 128.9, 124.4, 124.4, 116.3, 111.1, 61.2, 19.9, 14.6; **MS** (M.wt = 405.40), **(ESI positive)** *m/z* [M + H]^+^: 406.41; **LC/MS purity**: >95%.

#### Ethyl 2–(4-substituted benzamido)-1–(3-isopropoxypropyl)-1H-benzo[d]imidazole-5-carboxylate (VII and VIII)

*General procedure*: to the solution of ethyl 2-amino-1–(3-isopropoxypropyl)-1H-benzo[d]imidazole-5-carboxylate (**V**) (0.3 g, 1 mmol), the appropriate acid (1 mmol), TBTU (0.3 g, 2 mmol) and DMAP (0.64 g, 2 mmol) were added and dissolved in the least amount of DMF (15 ml) and allowed to stir under N_2_ atmosphere at room temperature for 24–48 h. The completion of the reactions was confirmed using TLC. The resulting solutions were poured on ice-cold water giving the titled compounds (**VII** and **VIII**) with yield of 60–70%. The titled compounds were purified by column chromatography using hexane: ethyl acetate gradient elution (9:1–1:1).

#### Ethyl 2–(4-chlorobenzamido)-1–(3-isopropoxypropyl)-1H-benzo[d]imidazole-5-carboxylate (VII)

The titled compound was separated as white crystals, yield 70%; m.p. 150–152 °C; **^1^H NMR (300 MHz, DMSO-d_6_)** δ 12.94 (s, 1H), 8.21 (d, *J* = 1.7 Hz, 2H), 8.15 (s, 1H), 7.91 (d, *J* = 1.5 Hz, 1H), 7.61 (m, 2H), 7.53 (m, 1H), 4.35 (m, 4H), 3.43 (m, 3H), 2.06 (m, 2H), 1.35 (t, *J* = 7.1 Hz, 3H), 1.01 (d, *J* = 6.1 Hz, 6H); **^13^C NMR (75 MHz, DMSO-d_6_)** δ 172.5, 166.1, 153.6, 140.6, 133.6, 133.3, 131.4, 130.5, 129.06, 128.8, 127.8, 124.7, 124.5, 71.0, 64.8, 61.2, 40.8, 28.8, 22.3, 14.7**; MS** (M.wt = 443.93), **(ESI positive)** *m/z* [M + H]^+^: 444.94; **LC/MS purity**: >95%.

#### Ethyl 2-(isonicotinamido)-1–(3-isopropoxypropyl)-1H-benzo[d]imidazole-5-carboxylate (VIII)

The titled compound was separated as yellowish white crystals, yield 75%; m.p. 148–150 °C; **^1^H NMR (300 MHz, DMSO-d_6_)** δ 13.00 (s, 1H), 8.75 (d, *J* = 5.9 Hz, 2H), 8.17 (s, 1H), 8.10 (d, *J* = 5.9 Hz, 2H), 7.92 (dd, *J* = 8.5, 1.6 Hz, 1H), 7.63 (d, *J* = 8.5 Hz, 1H), 4.51–4.23 (m, 4H), 3.47–3.37 (m, 3H), 2.05 (t, *J* = 6.2 Hz 2H), 1.35 (t, *J* = 7.1 Hz, 3H), 1.00 (d, *J* = 6.1 Hz, 6H); **^13^C NMR (75 MHz, DMSO-d_6_) δ** 172.4, 166.1, 153.6, 150.5, 145.3, 133.6, 129.3, 124.8, 124.7, 122.9, 113.7, 110.2, 71.0, 64.8, 61.2, 40.8, 28.8, 22.3, 14.7; **MS** (M.wt = 410.20), **(ESI positive)** *m/z* [M + H]^+^: 411.22; **LC/MS purity**: >95%.

#### Ethyl 1-alkyl-2-substituted-1H-benzo[d]imidazole-5-carboxylate (IX)

*General procedure:* To a solution of respective *N*-alkyl derivative (**III**) (1 equiv, 6.8 mmol) in DMSO (20 ml), benzaldehyde derivative (1.1 equiv.) and sodium dithionite (3.6 g, 20 mmol, 3 equivalents) were added. The reaction mixture was stirred at 90 °C for 7 h. After completion of the reaction, the reaction was poured onto (500 ml) ice-cold water with vigorous stirring where precipitate was formed. The precipitate was then filtered, washed with water and extracted with ethyl acetate to yield the respective derivatives of compounds (**IX**) with yield of (60–70%).

#### 1-Alkyl-2-substituted-1H-benzo[d]imidazole-5-carboxylic acid (X)

*General procedure:* ethyl 1-alkyl-2-substituted-1H-benzo[d]imidazole-5-carboxylate (**IX**) (1 equiv., 6.9 mmol) was added to a solution of LiOH.H_2_O (0.579 g, 13.8 mmol) in ethanol (50%, 70 ml). The mixture was heated under reflux for 2 h. The resulting solution was allowed to cool to room temperature then added to 10% HCl/ice (100 ml) with continued stirring. The resulting solid was filtered, washed with water and allowed to dry to afford the corresponding acid derivative (**X**) with yield about (85%).

#### 1-Alkyl-2-substituted-1H-benzo[d]imidazole-5-carboxamide derivative (XI and XII)

*General procedure:* a solution of the appropriate acid derivative (**X**) (1 equiv., 1 mmol), the appropriate amine (1 equiv., 1 mmol), TBTU (0.3 g, 2 mmol) and DMAP (0.64 g, 2 mmol) were dissolved in the least amount of DMF (15 ml) and allowed to stir under N_2_ atmosphere at room temperature for 24–48 h. The completion of the reactions was confirmed using TLC. The resulting solutions were poured on ice-cold water giving the titled compounds (**XI** and **XII**), of yield about (60–70%). The titled compounds were purified by column chromatography using hexane: ethyl acetate gradient elution (9:1–1:1) and confirmed by **^1^H NMR** spectroscopy.

#### 2-(Furan-2-yl)-1–(3-isopropoxypropyl)-N-(2,4,6-trimethoxyphenyl)-1H-benzo[d]imidazole-5-carboxamide (XI)

The titled compound was separated as white crystals, yield 80%; m.p. 138–140 °C; **^1^H NMR (300 MHz, DMSO-d_6_)** δ 10.12 (s, 1H), 8.52 (s, 1H), 8.35 (d, *J* = 1.6 Hz, 1H), 7.94 (d, *J* = 1.6 Hz, 1H), 7.91 (d, *J* = 1.6 Hz, 1H), 7.74 (d, *J* = 8.6 Hz, 1H), 7.31 (s, 2H), 7.15 (dd, *J* = 1.9, 0.8 Hz, 1H), 4.51 (t, *J* = 7.2 Hz, 2H), 3.73 (d, *J* = 41.2 Hz, 9H), 3.54–3.43 (m, 1H), 3.38 (t, *J* = 5.6 Hz, 2H), 2.14–1.82 (m, 2H), 1.08 (d, *J* = 6.1 Hz, 6H). **^13^C NMR (75 MHz, DMSO-d_6_)** δ 165.9, 153.0, 148.4, 144.7, 143.4, 142.5, 138.5, 136.1, 129.1, 122.5, 118.8, 116.8, 111.1, 110.6, 98.3, 71.3, 64.2, 56.2, 41.7, 30.3, 22.4; **MS** (M.wt = 493.22), **(ESI positive)** *m/z* [M + H]^+^: 494.25; **LC/MS purity**: >95%.

#### 1-Cyclohexyl-2–(4-methoxyphenyl)-*N*-(2,4,6-trimethoxyphenyl)-1H-benzo[d]imidazole-5-carboxamide (XII)

The titled compound is separated as white crystals, yield 75%; m.p. 138–140 °C; **^1^H NMR (300 MHz, DMSO-d_6_**) δ 10.13 (s, 1H), 8.38 (s, 1H), 7.96 (d, *J* = 8.7 Hz, 1H), 7.87 (dd, *J* = 8.6, 1.7 Hz, 1H), 7.62 (d, *J* = 8.7 Hz, 2H), 7.31 (s, 2H), 7.15 (d, *J* = 8.7 Hz, 2H), 3.86 (s, 6H), 3.78 (s, 6H), 2.31 (q, *J* = 11.9 Hz, 1H), 2.01–1.77 (m, 5H), 1.65 (d, *J* = 11.8 Hz, 1H), 1.42–1.28 (m, 4H);) . **^13^C NMR (75 MHz, DMSO-d_6_)** δ 165.9, 160.9, 155.3, 153.0, 143.3, 136.4, 133.9, 131.3, 128.7, 122.9, 122.2, 119.2, 114.7, 113.2, 98.4, 60.6, 56.2, 55.8, 31.0, 26.0; **MS** (M.wt = 515.24), **(ESI positive)** *m/z* [M + H]^+^: 516.22; **LC/MS purity**: >95%.

### Biological evaluation

#### Chemicals

Doxorubicin, rhodamine 123 (Rho123), and 3–(4,5-dimethylthiazol-2-yl)-2,5-diphenyl-tetrazolium bromide (MTT) were purchased from Sigma–Aldrich^®^ GmbH, Germany. DMEM and RPMI1640 media, non-essential amino acid (NEAA), sodium pyruvate, penicillin–streptomycin (P/S), foetal bovine serum (FBS), trypsin–EDTA, L-glutamine and dimethyl sulfoxide (DMSO) were obtained from Gibco^®^ Invitrogen, Germany.

#### Cell culture

Human T-cell lymphoma CCRF-CEM cells and the derived doxorubicin-resistant subline (CEM/ADR5000) were obtained from Professor T. Efferth, Department of Pharmaceutical Biology, University of Mainz, Germany. These suspension cells were cultured in RPMI1640 medium supplemented with 10% heat-inactivated calf serum, 2 mM L-glutamine, and antibiotics (penicillin, streptomycin). In order to maintain the high level of ABCB1transporters expression in CEM/ADR 5000 cells, they should be cultivated weekly for 24 h in with 5 µg/mL doxorubicin. Human breast adenocarcinoma) cell line MCF-7 was available in IPMB, Heidelberg University, Germany. Human epithelial colorectal adenocarcinoma cells (Caco-2) were bought from the German Collection of Microorganisms and Cell Cultures (DSMZ, Braunschweig, Germany). The adherent MCF-7 and Caco-2 cells were maintained in DMEM with glutamine (Invitrogen/Gibco^®^, Karlsruhe, Germany) supplemented with 10% FBS (foetal bovine serum) (BioChrom KG, Berlin, Germany), 100 U/mL penicillin, and 100 µg/mL streptomycin, 1% sodium pyruvate, and 1% NEAA. Adherent cells were detached from the culture vessel by adding 0.25% trypsin and 0.02% EDTA for 5 min. Cells were cultivated at 37 °C, 5% CO_2_, and 95% humidity. All experiments were repeated three times and performed with cells in the logarithmic growth phase.

#### Cytotoxicity (MTT) assay

The MTT assay was performed according to the reported method by Mosmann^44^ with minor modifications. For the suspension cells CCRF-CEM and CEM/ADR 5000, cells with a density of 2 x 10^4^ were seeded in 96-well plates, and various doses of the tested compounds were added. The plates were incubated for 48 h. The media were then removed, and 0.5% MTT (dissolved in media) was added and further incubated at 37 °C for 2–4 h. Afterwards, the plates were centrifuged at 400 rpm for 10 min. The formazan crystals were dissolved in 100 µL DMSO and the absorption was read at 570 nm with the Tecan Nano Quant infinite M200 PRO Plate Reader (Tecan, Männedorf, Switzerland). For adherent cell lines MCF-7 and Caco-2, cells were in the 96-well plates with a density of 2 x 10^4^ and 5 X 10^3^ cells, respectively, and incubated for 24 h at 37 °C. Subsequently, the media were removed and various doses of the target compounds prepared in media were added to the plates and incubated for 24 h (MCF-7) or 48 h (Caco-2). Then, the MTT solution (0.5 mg/mL) was added to all wells and incubated for another 4 h. The formed formazan crystals were dissolved in 100 µL DMSO and the absorption was read at 570 nm as previously mentioned. Doxorubicin was used as positive control. All experiments were repeated three times.

#### Effect of two drug combinations on cell viability

The combinations of target compounds with doxorubicin were investigated in doxorubicin resistant cells CEM/ADR 5000 and Caco-2 as previously described^45^. Cells were treated with doxorubicin together with different non-toxic concentrations (IC_10_, IC_20_ and IC_30_) of target compounds **VII**, **VIII** and **XI**. The seeded cells with the combinations were incubated for 24 h (CEM/ADR 5000) or 48 h (Caco-2); then the MTT assay was carried out as mentioned above.

#### Activity of ABC transporters in CEM/ADR 5000 cells

CEM/ADR5000 cells were seeded in the density of 1 × 10^4^ cells/mL in serum-free medium. The target compounds were added in various final concentrations (IC_50_, 50 µM and 100 µM), and incubated for 90 min at 37 °C. The cells were then washed twice with cold phosphate-buffered saline (PBS). Rho123 (10 µM final concentration) was added to the cells, and incubated for 90 min at 37 ^º^C. Again, the cells were washed twice, and resuspended in PBS for measurements using FACS tubes. Verapamil was used as a positive control and its competitive ABCB1 inhibitory activity was considered as 100%. The fluorescence intensity of Rho123 was measured by flow cytometry, using a Becton-Dickinson FACScan instrument, IPMB, Heidelberg University, Germany.

#### Statistical analyses

All assays were carried out in triplicates. All data are expressed as mean ± standard deviation. Graphs and data analysis of the cytotoxic assays were performed with SigmaPlot^®^ 11.0 (Systat Software, San Jose, CA, USA). The IC_50_ values were calculated from the dose–response curves using a four-parameter logistic fitting curve. Statistical significance was evaluated employing either one-way ANOVA or student paired t-test. A *p* values smaller than 0.05 was considered significant.

### In silico studies

#### Ligand based pharmacophore design and ligand pharmacophore mapping

Using Accelry’s Discovery Studio 2.5.5 software (Accelrys Inc., San Diego, CA, USA) at Faculty of Pharmacy, Ain Shams University, the reference compounds (**12**) and (**3**) were drawn in the 3 D-molecule window, and the essential pharmacophoric features for their selective activities were selected in each molecule (HD_Acceptor, Ring Aromatic, Hydrophobic) by manual feature mapping using features and location constrain in the query toolbar of the used software. The hybridised pharmacophore generated after combing features of both reference compounds was then used in mapping of the designed compounds using Ligand Pharmacophore mapping protocol, Accelry’s Discovery Studio 2.5.5 software. In the Protocols Explorer, expand the Pharmacophore folder and choose **ligand pharmacophore mapping**. In the input ligands parameter, the designed target benzimidazole compounds were selected. In the input pharmacophore parameter, the saved hybridised pharmacophore was selected, and then the protocol is run from the protocol toolbar. After the job was completed, the report was displayed and the mapped molecules were visualised and the resulted FitValues were compared.

#### Molecular modelling

Molecular modelling including docking and calculation of RMSD value was performed using Accelry’s Discovery Studio 2.5.5 software (Accelrys Inc., San Diego, CA, USA) at Faculty of Pharmacy, Ain Shams University. The X-ray crystal structure of ABCB1 co-crystallized with the reference inhibitor zosuquidar (**2**) was obtained from the Protein Data Bank at the Research Collaboration for Structural Bioinformatics (RCSB) website [https://www.rcsb.org] (PDB code: **6QEE**) and loaded in Accelry’s Discovery Studio. Docking of the target compounds together with the reference compounds (**2**) and (**3**) as ABCB1 inhibitors was done using CDOCKER protocol, Accelry’s Discovery Studio 2.5.5 software (Accelrys Inc., San Diego, CA, USA) at Faculty of Pharmacy, Ain Shams University. The CDOCKER interaction energies generated for the docked target compounds and their binding modes with the key aminoacids were compared to those of the reference compounds.

#### In vitro ADME study of the physiochemical properties of target compounds using lipiniski rule

The drugability of the cytotoxic target compounds was assessed using Molecular operating environment (MOE) software 2013 (Chemical Computing Group Inc., Montreal, QC, Canada) using Lipiniski rule protocol. The results were displayed as Lipiniski violoation. The compound is to be suggested oral bioavailable as the value approaches (0) which means that it fulfils the 5 parameters of the rule.

## Supplementary Material

Supplemental Material

## References

[1] Baguley BC. Multiple drug resistance mechanisms in cancer. Mol Biotechnol. 2010;46(3):308–316. 20717753 10.1007/s12033-010-9321-2

[2] Ambudkar SV, Dey S, Hrycyna CA, Ramachandra M, Pastan I, Gottesman MM. Biochemical, cellular, and pharmacological aspects of the multidrug transporter. Annu Rev Pharmacol Toxicol. 1999;39:361–398. 10331089 10.1146/annurev.pharmtox.39.1.361

[3] Eid SY, El-Readi MZ, Wink M. Carotenoids reverse multidrug resistance in cancer cells by interfering with ABC-transporters. Phytomedicine. 2012;19(11):977–987. 22770743 10.1016/j.phymed.2012.05.010

[4] Szakács G, Paterson JK, Ludwig JA, Booth-Genthe C, Gottesman MM. Targeting multidrug resistance in cancer. Nat Rev Drug Discov. 2006;5(3):219–234. 16518375 10.1038/nrd1984

[5] Chen L, Li Y, Yu H, Zhang L, Hou T. Computational models for predicting substrates or inhibitors of P-glycoprotein. Drug Discov Today. 2012;17(7-8):343–351. 22119877 10.1016/j.drudis.2011.11.003

[6] Choi CH. ABC transporters as multidrug resistance mechanisms and the development of chemosensitizers for their reversal. Cancer Cell Int. 2005;5:30. 16202168 10.1186/1475-2867-5-30 PMC1277830

[7] Gatti L, Cossa G,L, Beretta G, Zaffaroni N, Perego P. Novel insights into targeting ATP-binding cassette transporters for antitumor therapy. Curr Med Chem. 2011;18(27):4237–4249. 21838682 10.2174/092986711797189682

[8] Choi Y, Yu A-M. ABC transporters in multidrug resistance and pharmacokinetics, and strategies for drug development. Curr Pharm Des. 2014;20(5):793–807. 23688078 10.2174/138161282005140214165212 PMC6341993

[9] Robey RW, Pluchino KM, Hall MD, Fojo AT, Bates SE, Gottesman MM. Revisiting the role of ABC transporters in multidrug-resistant cancer. Nat Rev Cancer. 2018;18(7):452–464. 29643473 10.1038/s41568-018-0005-8 PMC6622180

[10] Wang S, Wang S-Q, Teng Q-X, Yang L, Lei Z-N, Yuan X-H, Huo J-F, Chen X-B, Wang M, Yu B, et al. Structure-based design, synthesis, and biological evaluation of new triazolo[1,5- a]pyrimidine derivatives as highly potent and orally active ABCB1 modulators. J Med Chem. 2020;63(24):15979–15996. 33280384 10.1021/acs.jmedchem.0c01741

[11] Tsuruo T, Iida H, Tsukagoshi S, Sakurai Y. Overcoming of vincristine resistance in P388 leukemia in vivo and in vitro through enhanced cytotoxicity of vincristine and vinblastine by verapamil. Cancer Res. 1981;41(5):1967–1972. 7214365

[12] Cripe LD, Uno H, Paietta EM, Litzow MR, Ketterling RP, Bennett JM, Rowe JM, Lazarus HM, Luger S, Tallman MS, et al. Zosuquidar, a novel modulator of P-glycoprotein, does not improve the outcome of older patients with newly diagnosed acute myeloid leukemia: a randomized, placebo-controlled trial of the Eastern Cooperative Oncology Group 3999. Blood. 2010;116(20):4077–4085. 20716770 10.1182/blood-2010-04-277269 PMC2993615

[13] Palmeira A, Sousa E,H, Vasconcelos M,M, Pinto M. Three decades of P-gp inhibitors: skimming through several generations and scaffolds. Curr Med Chem. 2012;19(13):1946–2025. 22257057 10.2174/092986712800167392

[14] Gottesman MM, Fojo T, Bates SE. Multidrug resistance in cancer: role of ATP-dependent transporters. Nat Rev Cancer. 2002;2(1):48–58. 11902585 10.1038/nrc706

[15] Chen T, Wang C, Liu Q, Meng Q, Sun H, Huo X, Sun P, Peng J, Liu Z, Yang X, et al. Dasatinib reverses the multidrug resistance of breast cancer MCF-7 cells to doxorubicin by downregulating P-gp expression via inhibiting the activation of ERK signaling pathway. Cancer Biol Ther. 2015;16(1):106–114. 25482933 10.4161/15384047.2014.987062 PMC4622436

[16] Baumert C, Hilgeroth A. Recent advances in the development of P-gp inhibitors. Anticancer Agents Med Chem. 2009;9(4):415–436. 19442042 10.2174/1871520610909040415

[17] Maiti B, Chanda K. Diversity oriented synthesis of benzimidazole-based biheterocyclic molecules by combinatorial approach: a critical review. RSC Adv. 2016;6(56):50384–50413.

[18] Lissitchkov T, Arnaudov G, Peytchev D, Merkle K. Phase-I/II study to evaluate dose limiting toxicity, maximum tolerated dose, and tolerability of bendamustine HCl in pre-treated patients with B-chronic lymphocytic leukaemia (Binet stages B and C) requiring therapy. J Cancer Res Clin Oncol. 2006;132(2):99–104. 16292542 10.1007/s00432-005-0050-z PMC12161039

[19] Gandhi V, Burger JA. Bendamustine in B-cell malignancies: the new 46-year-old kid on the block. Clin Cancer Res. 2009;15(24):7456–7461. 19996200 10.1158/1078-0432.CCR-08-3041 PMC2795094

[20] Leoni LM, Bailey B, Reifert J, Bendall HH, Zeller RW, Corbeil J, Elliott G, Niemeyer CC. Bendamustine (Treanda) displays a distinct pattern of cytotoxicity and unique mechanistic features compared with other alkylating agents. Clin Cancer Res. 2008;14(1):309–317. 18172283 10.1158/1078-0432.CCR-07-1061

[21] Ghasemi F, Black M, Vizeacoumar F, Pinto N, Ruicci KM, Le CCSH, Lowerison MR, Leong HS, Yoo J, Fung K, et al. Repurposing Albendazole: new potential as a chemotherapeutic agent with preferential activity against HPV-negative head and neck squamous cell cancer. Oncotarget. 2017;8(42):71512–71519. 29069723 10.18632/oncotarget.17292 PMC5641066

[22] Yenjerla M, Cox C, Wilson L, Jordan MA. Carbendazim inhibits cancer cell proliferation by suppressing microtubule dynamics. J Pharmacol Exp Ther. 2009;328(2):390–398. 19001156 10.1124/jpet.108.143537 PMC2682274

[23] Dillman RO, Koziol JA. Phase I cancer trials: limitations and implications. Mol Biother. 1992;4(3):117–121. 1445664

[24] Al-Douh MH, Sahib HB, Osman H, Hamid SA, Salhimi SM. Anti-proliferation effects of benzimidazole derivatives on HCT-116 colon cancer and MCF-7 breast cancer cell lines. Asian Pac J Cancer Prev. 2012;13(8):4075–4079. 23098519 10.7314/apjcp.2012.13.8.4075

[25] Abdel-Aziz HA, Ghabbour HA, Eldehna WM, Al-Rashood STA, Al-Rashood KA, Fun H-K, Al-Tahhan M, Al-Dhfyan A. 2-((Benzimidazol-2-yl)thio)-1-arylethan-1-ones: Synthesis, crystal study and cancer stem cells CD133 targeting potential. Eur J Med Chem. 2015;104:1–10. 26413725 10.1016/j.ejmech.2015.09.023

[26] Yoon YK, Ali MA, Wei AC, Choon TS, Shirazi AN, Parang K. Discovery of a potent and highly fluorescent sirtuin inhibitor. Med Chem Commun. 2015;6(10):1857–1863.

[27] Sydnes MO, Isobe M. Synthesis of the second generation photoaffinity probes of tautomycin. Tetrahedron. 2007;63(12):2593–2603.

[28] Kuramoto Y, Ohshita Y, Yoshida J, Yazaki A, Shiro M, Koike T. A novel antibacterial 8-chloroquinolone with a distorted orientation of the N1-(5-amino-2,4-difluorophenyl) group. J Med Chem. 2003;46(10):1905–1917. 12723953 10.1021/jm0205090

[29] WO2008130368A2 - Transcription factor modulating compounds and methods of use thereof - Google Patents. 2021. https://patents.google.com/patent/WO2008130368A2/en

[30] Lemus RH, Lee CH, Skibo EB. Studies of extended quinone methides. Synthesis and physical studies of purine-like monofunctional and bifunctional imidazo[4,5-g]quinazoline reductive alkylating agents. J Org Chem. 1989;54(15):3611–3618.

[31] Ampati S, Vidyasagar JV, Swathi K, Sarangapani M. Synthesis and invitro evaluation of novel benzoxazole derivatives as specific cyclooxygenase-2 inhibitors. J Chem Pharm Res. 2010;2:213–219.

[32] González-Álvarez M, Alzuet G, Borrás J, Del Castillo Agudo L, García-Granda S, Montejo Bernardo JM. Strong protective action of Copper(II) N-substituted sulfonamide complexes against reactive oxygen species. J Inorg Biochem. 2004;98(2):189–198. 14729299 10.1016/j.jinorgbio.2003.10.012

[33] Han X, Zhong Y, Zhou G, Qi H, Li S, Ding Q, Liu Z, Song Y, Qiao X. Synthesis and biological evaluation of N-(carbobenzyloxy)-L-phenylalanine and N-(carbobenzyloxy)-L-aspartic acid-β-benzyl ester derivatives as potent topoisomerase IIα inhibitors. Bioorg Med Chem. 2017;25(12):3116–3126. 28462840 10.1016/j.bmc.2017.03.065

[34] Oda S, Shimizu H, Aoyama Y, Ueki T, Shimizu S, Osato H, Takeuchi Y. Development of safe one-pot synthesis of N-1- and C-2-substituted benzimidazole via reductive cyclization of o-nitroarylamine using Na 2S 2O 4. Org Process Res Dev. 2012;16(1):96–101.

[35] Chen KX, Njoroge FG, Pichardo J, Prongay A, Butkiewicz N, Yao N, Madison V, Girijavallabhan V. Design, synthesis, and biological activity of m-tyrosine-based 16- and 17-membered macrocyclic inhibitors of hepatitis C virus NS3 serine protease. J Med Chem. 2005;48(20):6229–6235. 16190750 10.1021/jm050323b

[36] Gibson CL, Huggan JK, Kennedy A, Kiefer L, Lee JH, Suckling CJ, Clements C, Harvey AL, Hunter WN, Tulloch LB, et al. Diversity oriented syntheses of fused pyrimidines designed as potential antifolates. Org Biomol Chem. 2009;7(9):1829–1842. 19590778 10.1039/b818339b

[37] Cortés-Funes H, Coronado C. Role of anthracyclines in the era of targeted therapy. Cardiovasc Toxicol. 2007;7(2):56–60. 17652804 10.1007/s12012-007-0015-3

[38] Shen H, Xu W, Chen Q, Wu Z, Tang H, Wang F. Tetrandrine prevents acquired drug resistance of K562 cells through inhibition of mdr1 gene transcription. J Cancer Res Clin Oncol. 2010;136(5):659–665. 19898868 10.1007/s00432-009-0704-3 PMC11828089

[39] Carvalho C, Santos RX, Cardoso S, Correia S, Oliveira PJ, Santos MS, Moreira PI. Doxorubicin: the good, the bad and the ugly effect. Curr Med Chem. 2009;16(25):3267–3285. 19548866 10.2174/092986709788803312

[40] Kadioglu O, Cao J, Kosyakova N, Mrasek K, Liehr T, Efferth T. Genomic and transcriptomic profiling of resistant CEM/ADR-5000 and sensitive CCRF-CEM leukaemia cells for unravelling the full complexity of multi-factorial multidrug resistance. Sci Rep. 2016;6(1):18. 27824156 10.1038/srep36754 PMC5099876

[41] Chou TC. Drug combination studies and their synergy quantification using the chou-talalay method. Cancer Res. 2010;70(2):440–446. 20068163 10.1158/0008-5472.CAN-09-1947

[42] Lipinski CA, Lombardo F, Dominy BW, Feeney PJ. Experimental and computational approaches to estimate solubility and permeability in drug discovery and development settings. Adv Drug Deliv Rev. 2001;46(1-3):3–26. 11259830 10.1016/s0169-409x(00)00129-0

[43] Benet LZ, Hosey CM, Ursu O, Oprea TI. BDDCS, the Rule of 5 and drugability. Adv Drug Deliv Rev. 2016;101:89–98. 27182629 10.1016/j.addr.2016.05.007 PMC4910824

[44] Mosmann T. Rapid colorimetric assay for cellular growth and survival: Application to proliferation and cytotoxicity assays. J Immunol Methods. 1983;65(1-2):55–63. 6606682 10.1016/0022-1759(83)90303-4

[45] Eid SY, El-Readi MZ, Wink M. Digitonin synergistically enhances the cytotoxicity of plant secondary metabolites in cancer cells. Phytomedicine. 2012;19(14):1307–1314. 23062361 10.1016/j.phymed.2012.09.002

