# Supplementary material for: Molecular design, synthesis and biological evaluation of novel 1,2,5-trisubstituted benzimidazole derivatives as cytotoxic agents endowed with ABCB1 inhibitory action to overcome multidrug resistance in cancer cells
Source: J Enzyme Inhib Med Chem. 2022 Sep 27;37(1):2710–24. doi: 10.1080/14756366.2022.2127700 (PMC9543179; doi:10.1080/14756366.2022.2127700)

Molecular design, synthesis and biological evaluation of novel 1,2,5-trisubstituted benzimidazole derivatives as cytotoxic agents endowed with ABCB1 inhibitory action to overcome Multidrug Resistance in cancer cells

Results and Discussion

Biological Evaluation

Dose response curve in CCRF/CEM cells

Doxorubicin

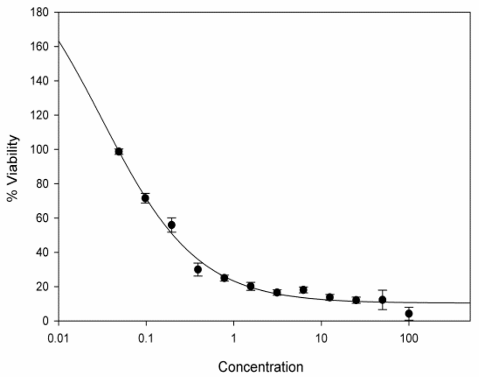

IC_{50} = 0.20 µM

Compound **VII**

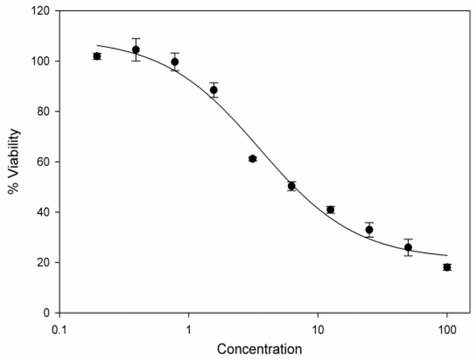

IC_{50} = 6.63 µM

Compound **VIII**

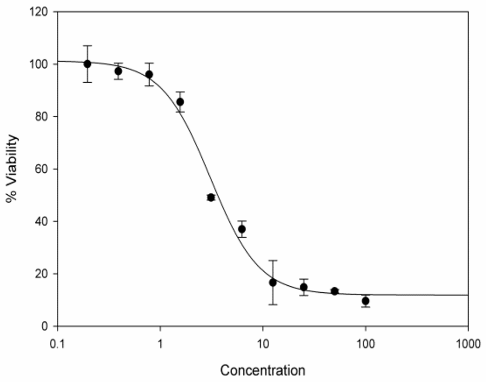

IC_{50} = 3.61 µM

Compound **XI**

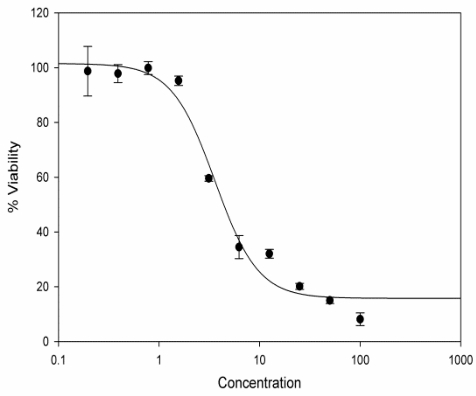

IC_{50} = 4.45 µM

Dose response curve in MCF7 cells

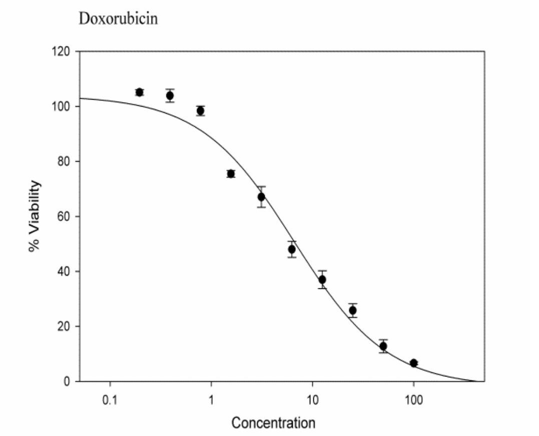

IC_{50} = 6.91 µM

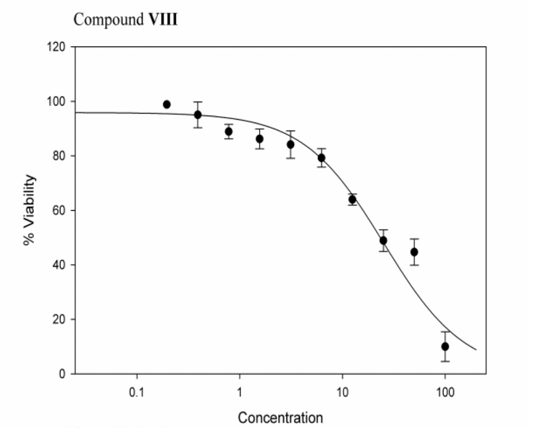

IC_{50} = 23.30 µM

Dose response curve in CEM/ADR5000 cells

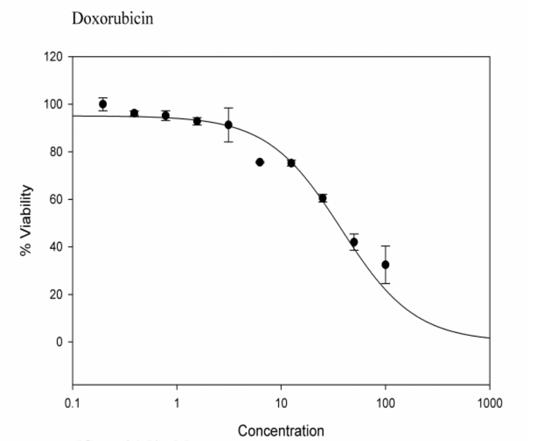

IC_{50} = 34.50 µM

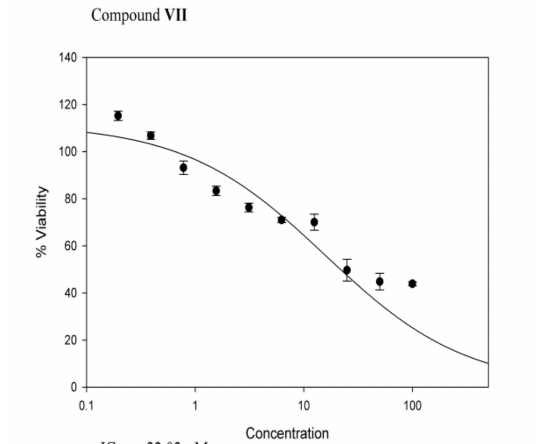

IC_{50} = 22.02 µM

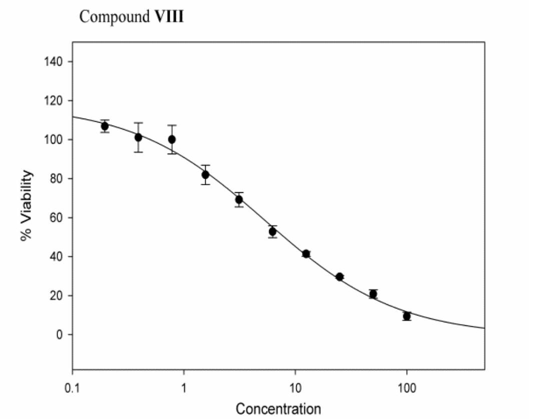

IC_{50} = 8.13 µM

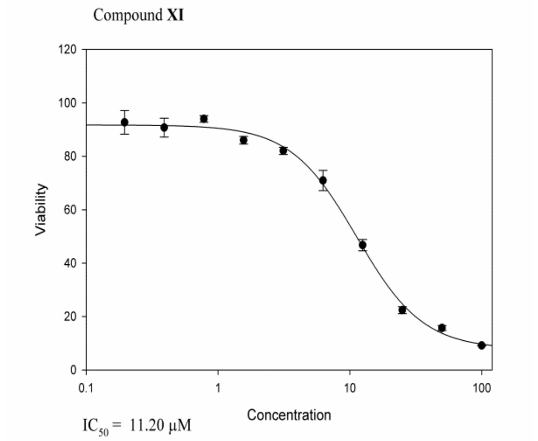

IC_{50} = 11.20 µM

Dose response curve in Caco-2 cells

Doxorubicin

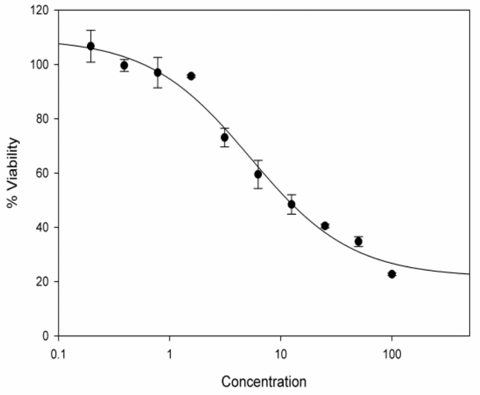

IC_{50} = 11.69 µM

Compound **VII**

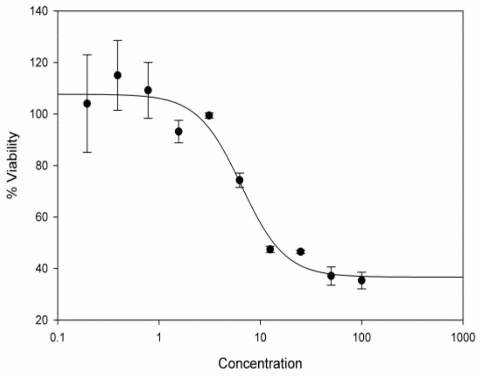

IC_{50} = 13.47 µM

Compound **VIII**

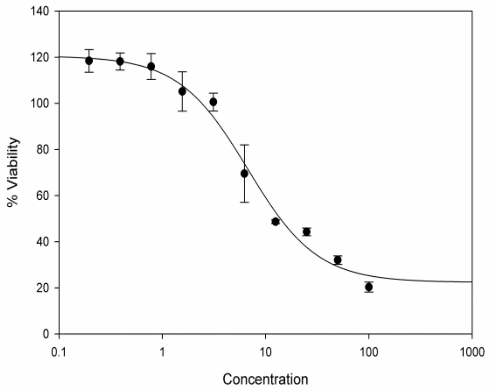

IC_{50} = 13.86 µM

Experimental

Chemistry and analysis

Supplementary Data NMR charts

Ethyl 1-acetyl-2-((4-fluorophenyl)sulfonamido)-1H-benzo[d]imidazole-5-carboxylate (VI)

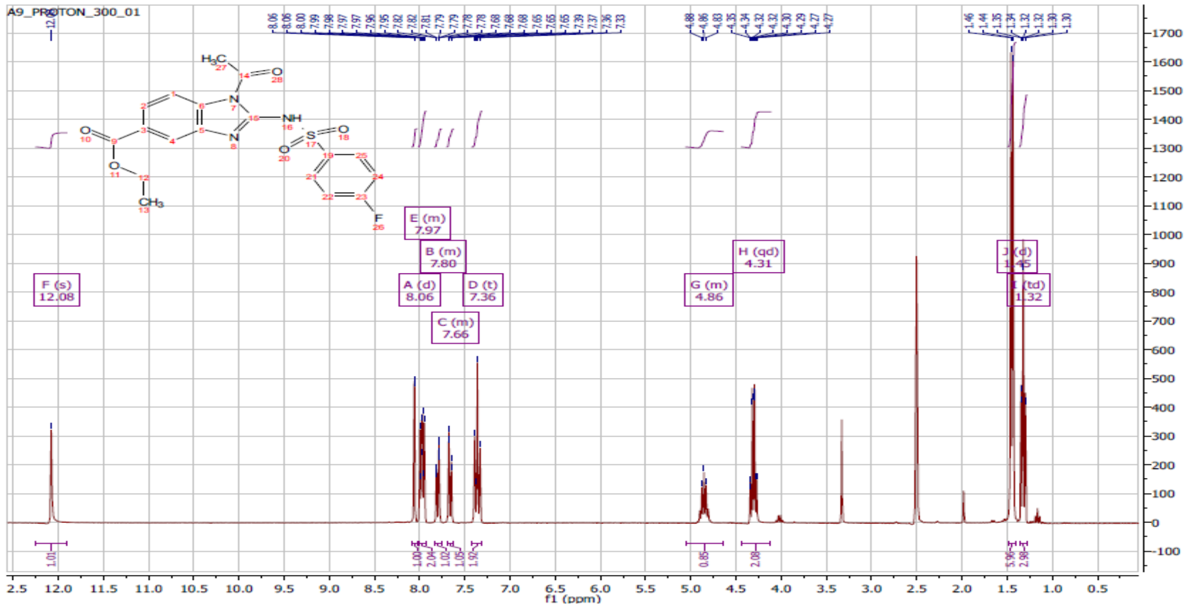

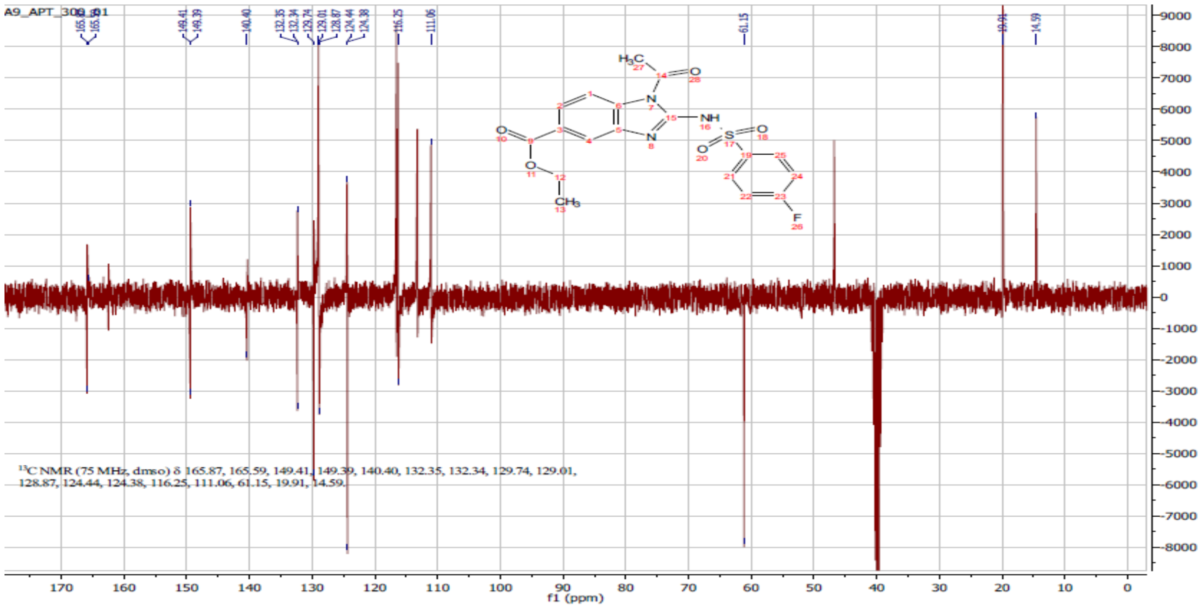

Ethyl 2-(4-chlorobenzamido)-1-(3-isopropoxypropyl)-1H-benzo[d]imidazole-5-carboxylate (VII)

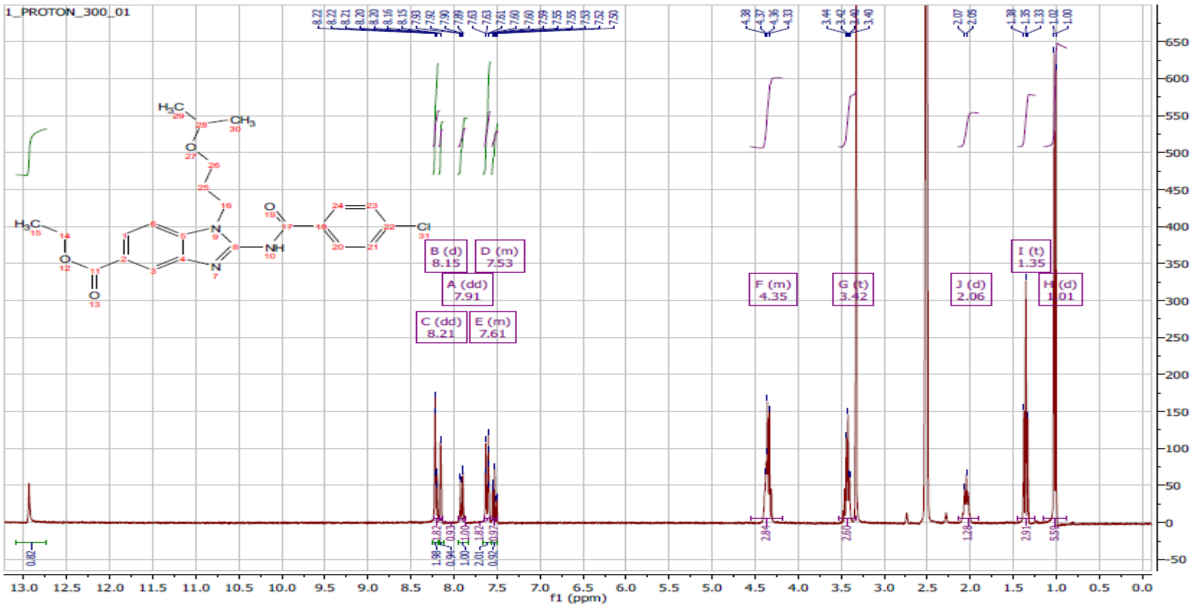

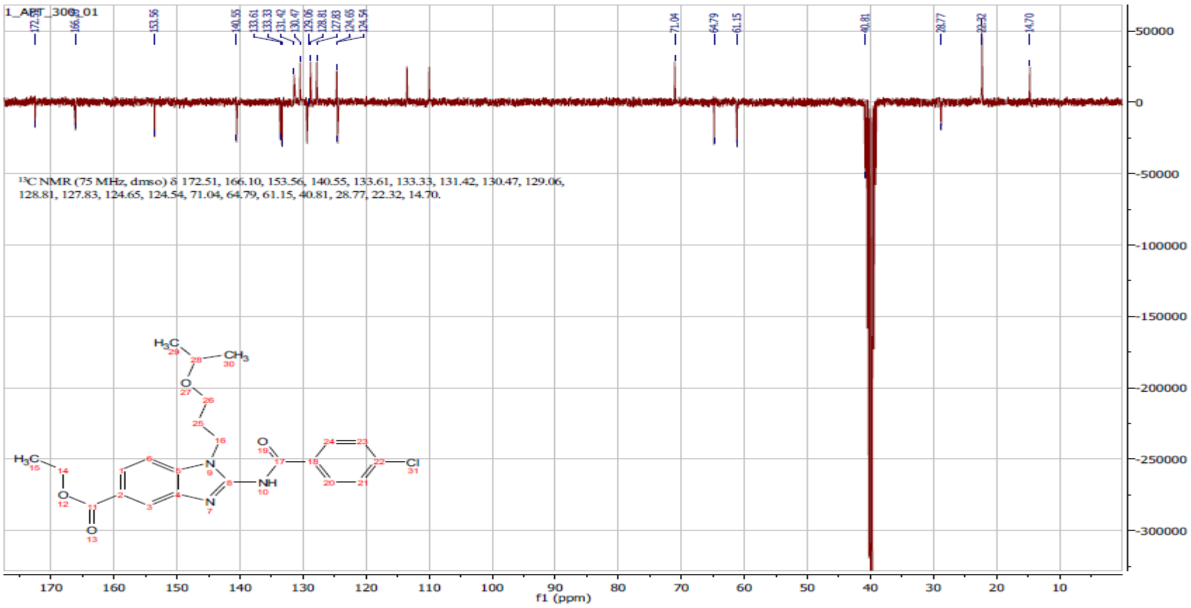

Ethyl 2-(isonicotinamido)-1-(3-isopropoxypropyl)-1H-benzo[d]imidazole-5-carboxylate (VIII)

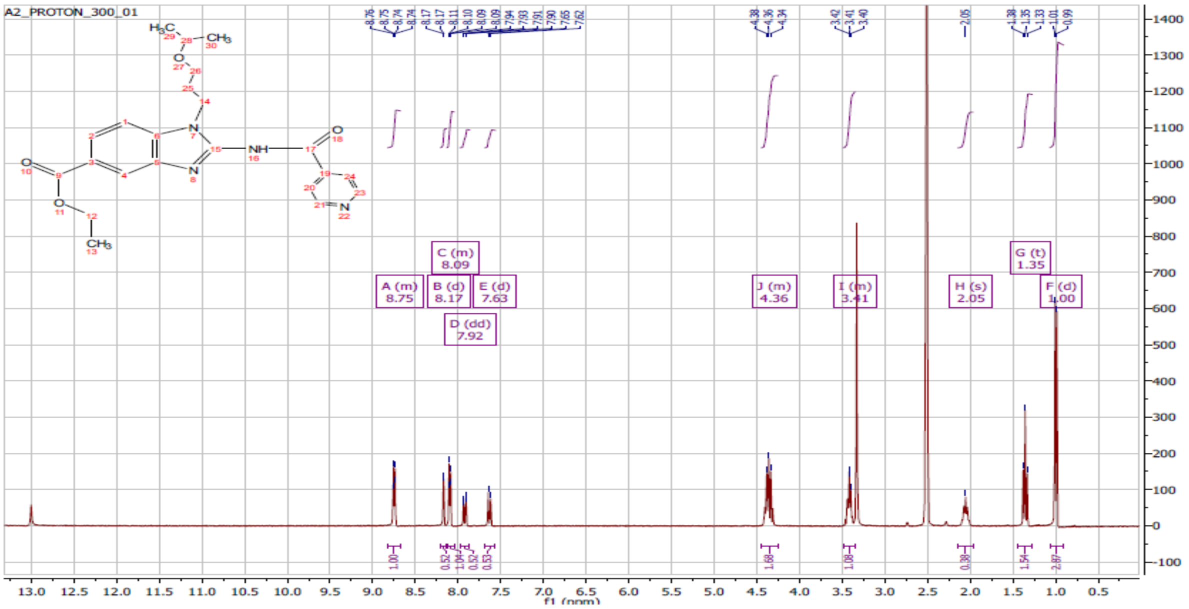

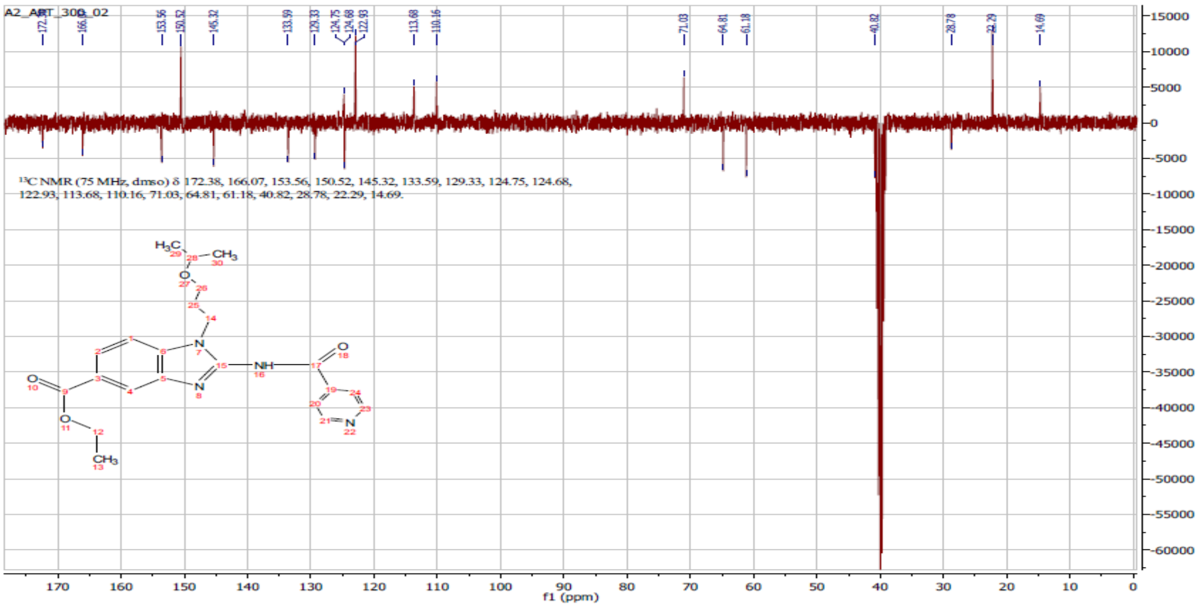

2-(Furan-2-yl)-1-(3-isopropoxypropyl)-N-(2,4,6-trimethoxyphenyl)-1H-benzo[d]imidazole-5-carboxamide (XI)

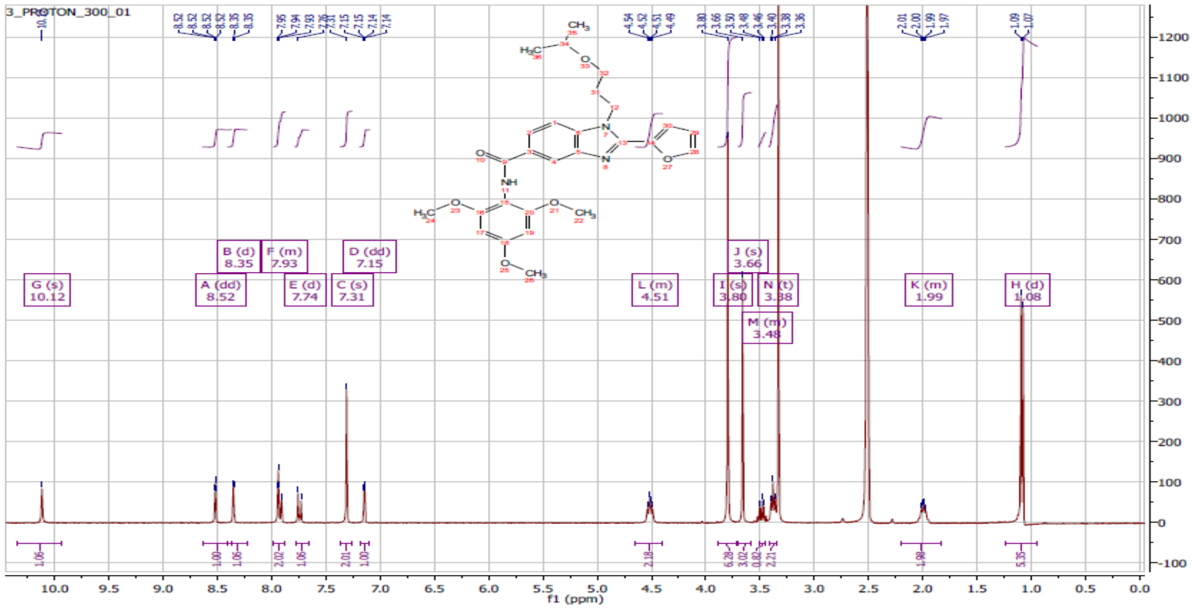

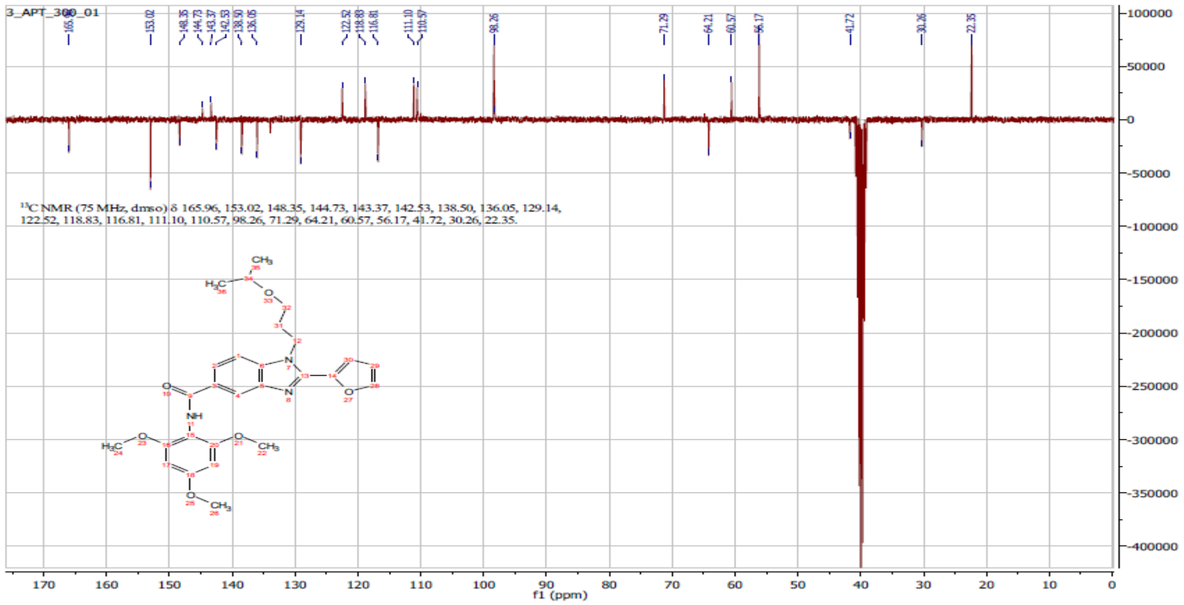

1-Cyclohexyl-2-(4-methoxyphenyl)-N-(2,4,6-trimethoxyphenyl)-1H-benzo[d]imidazole-5-carboxamide (XII)

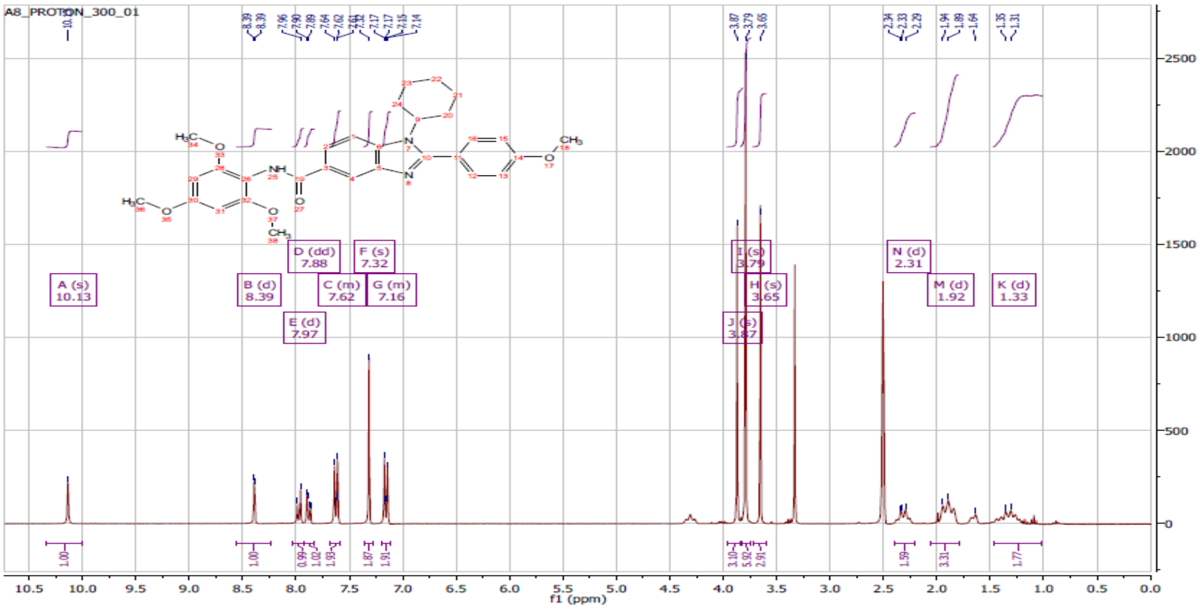

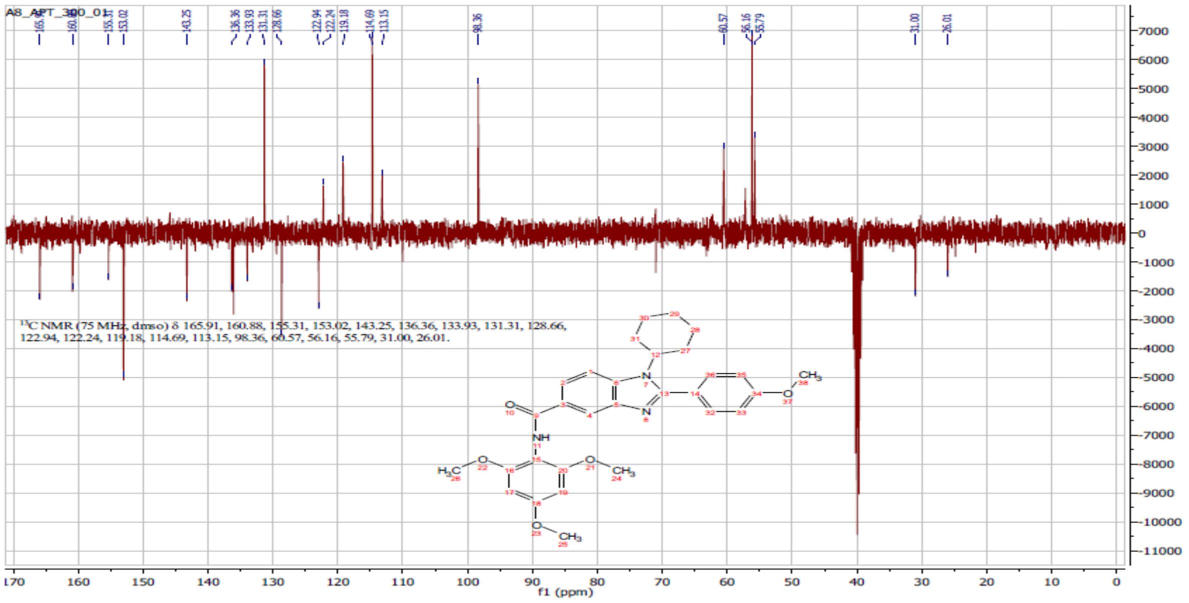

Supplement: Supplemental Material [file IENZ_A_2127700_SM4314.pdf]
